# Investigating dye performance and crosstalk in fluorescence enabled bioimaging using a model system

**DOI:** 10.1371/journal.pone.0188359

**Published:** 2017-11-27

**Authors:** Riikka Arppe, Miguel R. Carro-Temboury, Casper Hempel, Tom Vosch, Thomas Just Sørensen

**Affiliations:** 1 Nano-Science Center & Department of Chemistry, University of Copenhagen, Copenhagen, Denmark; 2 Department of Micro- and Nanotechnology, Technical University of Denmark, Kgs Lyngby, Denmark; Oregon State University, UNITED STATES

## Abstract

Detailed imaging of biological structures, often smaller than the diffraction limit, is possible in fluorescence microscopy due to the molecular size and photophysical properties of fluorescent probes. Advances in hardware and multiple providers of high-end bioimaging makes comparing images between studies and between research groups very difficult. Therefore, we suggest a model system to benchmark instrumentation, methods and staining procedures. The system we introduce is based on doped zeolites in stained polyvinyl alcohol (PVA) films: a highly accessible model system which has the properties needed to act as a benchmark in bioimaging experiments. Rather than comparing molecular probes and imaging methods in complicated biological systems, we demonstrate that the model system can emulate this complexity and can be used to probe the effect of concentration, brightness, and cross-talk of fluorophores on the detected fluorescence signal. The described model system comprises of lanthanide (III) ion doped Linde Type A zeolites dispersed in a PVA film stained with fluorophores. We tested: F18, MitoTracker Red and ATTO647N. This model system allowed comparing performance of the fluorophores in experimental conditions. Importantly, we here report considerable cross-talk of the dyes when exchanging excitation and emission settings. Additionally, bleaching was quantified. The proposed model makes it possible to test and benchmark staining procedures before these dyes are applied to more complex biological systems.

## Introduction

Imaging biological samples requires a diverse methodological skill set bridging microscopy, fluorescence, dye chemistry, sample preparation, staining and image analysis, and most importantly in-depth biological or medical knowledge to formulate and address a hypothesis. Bioimaging is a complicated task even when disregarding the heterogeneous and complex biological system.[1–4] Considering fluorescence microscopy exclusively, a variety of methods and commercial implementations exist.[4–10] Often the bioimaging experiment will be defined by the microscopes and imaging methods available in a given lab. The different equipment alone makes data recorded in one lab difficult to reproduce in a different lab, and this is a major problem since direct comparison of experimental results is essential. When the complexity of the biological system is factored in, along with the locally developed sample preparation and staining procedures, data reproduction becomes even more challenging. Here, we propose a model system that will make it possible to benchmark the microscope, the method, the mix of fluorescent probes, and the image analysis employed in a specific experiment. The model system can be readily recreated in different labs, thus differences arising due to the microscope, method and the chosen molecular probes can be explored experimentally and eliminated.

Fluorescence microscopy owes its strength to the unique ability to differentiate between the signal and background. This is achieved using fluorescent probes, and relies on the fact that the detected fluorescent signal arises from the administered probes. The fluorescent signal depends on the selected probe (brightness and color), the sample preparation (dye loading), the microscope (illumination, optics, detector), and the image analysis (background subtraction *etc*.). Furthermore, fluorescence signal of the molecular probes may vary depending on local interaction with the heterogeneous surrounding (e.g. quenching, increased emission intensity, spectral shifts, changed photostability *etc*.). All these factors have to be considered when comparing data from one imaging experiment to another. In particular, dye loading, microscopy hardware and the selected settings vary, which greatly influences the raw data, while the fluorescent signal is heavily influenced by the subsequent data analysis.[11–14]

Great accomplishments have been made in developing new and improved fluorescent probes.[15–27] These fluorophores have been developed and benchmarked on equal terms, which makes sense when comparing their photophysical properties. But when considering an imaging experiment, the dyes should be compared in the actual conditions used. Excitation intensity and wavelength, efficiency of optics and detectors, and in particular the background/autofluorescence of a given system, as well as quenching and photobleaching can be much more important for the outcome of a bioimaging experiment than the intrinsic properties of the dye. Fluorophores with good photophysical properties usually make for good probes, but further developments are required to guarantee that fluorophores perform well in a heterogeneous system under a microscope.[18, 25, 28] Therefore, we suggest a model system for comparing fluorescent probes in a controlled environment, and in the actual microscope used with realistic settings.

As many biological samples are inherently fluorescent, we must ensure that our signal is from our molecular probe and not the background. To do so often an excess of the chosen dye is added to the system. In a multicolor/multiprobe experiment this might lead to overloading of the system and to crosstalk between channels. All these issues can be probed using a model system containing similar dye concentrations and imaged in the actual microscope.

Contrast is the difference between fluorescent signal and background signal in the final image; this is the critical parameter in bioimaging. The end goal is perfect contrast, ideally where images are recorded with full knowledge of origin of photons. Imaging then becomes binary; is there probe in the pixel or not. It is not possible to compare the signal to background ratio achieved with different methods, when they are obtained using vastly different experimental conditions. In particular, the differences that occur in staining methods are not readily comparable. Therefore, we suggest that a common model sample is used for method comparisons. The benchmarked methods can then be used for comparing staining procedures and different molecular probes. At some point, the unstandardized approaches often applied in bioimaging must give way to systematic methodologies and common staining protocols. By establishing model systems for method comparison we can take the first step in this direction. The model system we propose here is not perfect, but is useful in this context. We used zeolites and a polymer thin film dyed with fluorophores as a model system for comparing contrast (signal recovered by subtracting the background from the raw data) in fluorescence microscopy. Bright fluorescent probes are homogeneously distributed in the polyvinyl alcohol (PVA) matrix, while lanthanide centered emitters in the zeolites act as reference points and as a potential method benchmark.

**Fig 1:**
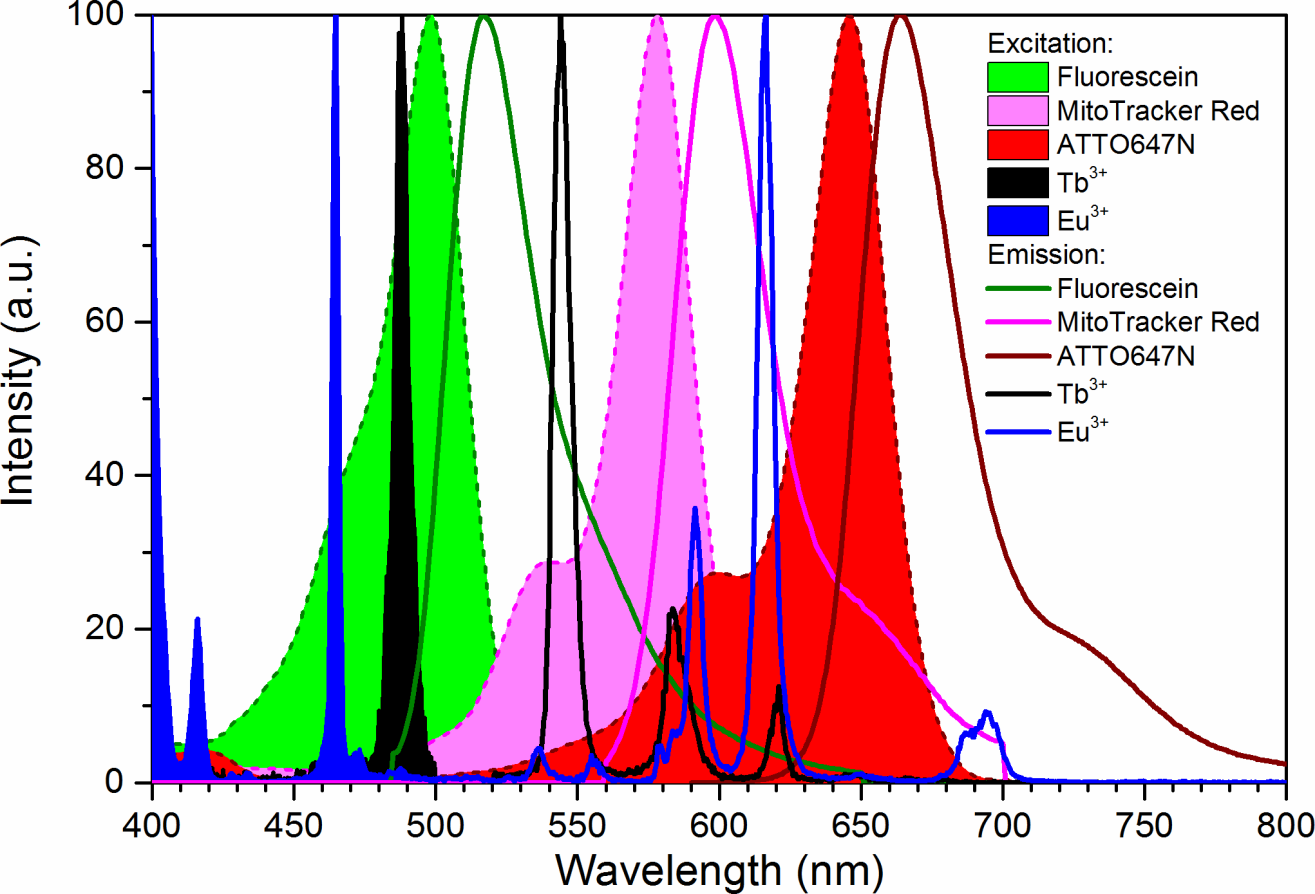
Spectral properties of chosen dyes. Normalized excitation (filled) and emission (lines) spectra of F18 (green), MitoTracker Red (magenta), ATTO647N (red), Tb(III) (black), and Eu(III) (blue).

In this work we combine multiple dyes that are used frequently in bioimaging. We used F18, MitoTracker Red, and ATTO647N. The excitation and emission spectra of these are shown in Fig 1, along with the spectra of the lanthanide(III) ions used to dope the zeolites. We demonstrate the relevance of a model system, by comparing bleaching, the achieved fluorescent signal intensity, and crosstalk for one specific set of imaging parameters and arbitrarily chosen concentration ratios. We found that the images in these experimental conditions can lead to unexpected contrast and cross-talk problems, which lead us to the conclusion that using a common benchmark to compare dye performance is highly relevant.

## Methods and materials

Fluorescein F18 was from Sigma-Aldrich (St. Louis, MO, US), MitoTracker Red (CMXRos) from Molecular Probes (ThermoFisher Scientific, Waltham, MA) and ATTO647N from ATTO-TEC GmbH (Siegen, Germany). Tb(III) acetate hydrate (99.9%), and Eu(III) acetate hydrate (99.9%) were purchased from Sigma-Aldrich (St. Louis, MO, US). Linde Type 5A (LTA) zeolites were a gift from UOP Antwerpen. Poly(vinyl alcohol) (PVA, 98% hydrolyzed, average M_w_ 13 000–23 000) was from Sigma-Aldrich.

### Model system

Our model system consisted of lanthanide(III) ions doped in zeolites and a polymer thin film dyed with fluorophores. The Ca^2+^ cations inside the pores and cavities of Linde Type A (LTA) zeolite were first exchanged with Eu(III) or Tb(III) cations by mixing 200 mg of zeolite in 800 μl of 0.25 M Ln(III) acetate hydrate in milliQ-water over night at room temperature (Vortex 3, IKA, Staufen, Germany). The Ln(III) exchanged zeolites were recovered and washed with 1 ml of milliQ-water thrice by centrifugation (1000 rpm, 2 min, Force 12, Denver Instrument, Bohemia, NY) and finally dispersed into 1 ml of MQ-water. The polymer thin film was formed by mixing 2 mg of Eu(III)@LTA and 2 mg of Tb(III)@LTA with 3% (w/v) PVA dyed with either 150 μM of F18 (5-octadecanoyl amino-fluorescein), 0.1 μM of MitoTracker Red, or 0.1 μM ATTO647N, and 50 μl of this mixture was spin-coated (SCI-10, Novocontrol, Montabaur, Germany) using dynamic dispense for ~1 min at ≈4000 rpm on a 22 × 22 mm microscope cover slip (Menzel-Gläser #1.5). Prior to use, the microscope slides were cleaned by pyrolysis at 450°C for a minimum of 1 hour.

### Confocal fluorescence microscope

A SuperK EXTREME EXB-6 supercontinuum white light laser with a SuperK SELECT wavelength selector (NKT Photonics, Birkerød, Denmark) was used as the excitation source. Four excitation wavelengths were selected: 465 nm, 488 nm, 560 nm, and 633 nm, for Eu(III), Tb(III) and F18, MitoTracker Red, and ATTO647N, respectively. The laser powers with 77.88 MHz repetition rate for each wavelength were 2 μW, 7.2 μW, 1.2 μW and 2.9 μW, respectively. Shortpass or bandpass filters (Table 1) were added to the excitation light path.

**Table 1:** Excitation parameters and filters used for microscopy.

Dye	Excitation wavelength	Excitation power	Excitation filter	Emission filter
Eu(III)	465 nm	2 μW	540 SP (540AESP, Omega optical)	532LP 2× (BLP01-532R-25, Semrock)
Tb(III), F18	488 nm	7.2 μW	540 SP (540AESP, Omega optical)	532LP 2× (BLP01-532R-25, Semrock)
MitoTracker Red	560 nm	1.2 μW	561 BP (LL02-561-25, Semrock)	560LP 2× (BLP01-561R-25, Semrock)
ATTO647N	633 nm	2.9 μW	633 BP (LL01-633-25, Semrock)	647LP (BLP01-647R-25, Semrock)

SP, short pass; LP, long pass; BP, band pass

The home-built scanning fluorescence confocal microscopy setup was based on an Olympus IX71 inverted microscope with a piezo-driven scanning stage (P5173CL, Physik Intrumente, Karlsruhe, Germany), controlled by a home-written software program (LabView, National Instruments), allowing for point-by-point imaging of the sample in a raster scanning fashion in a range up to 100 μm × 100 μm. Upon laser illumination, the emission signal from the sample was collected by the same 100× oil immersion objective (Olympus UPLFLN 100×, 1.3 NA). A 70/30 beamsplitter (XF122, Omega Filters) was used in microscope instead of a dichroic mirror.

The emission light was focused though a 50 μm pinhole, directed through optical long pass filters (Table 1) and detected in a CCD-based spectrometer (Princeton Instruments SPEC-10:100B/LN_eXcelon CCD camera, SP 2356 spectrometer with 1-030-500 grating 300 g/mm @ 500 nm, all controlled by the same LabView program that controls the scanner). The X axis of the emission spectra was calibrated using emission lines of a neon lamp (6032 neon lamp, Newport Corporation, Irvine, CA). The Y axis (Intensity) was not corrected for differences in optical transmission and detection efficiency.

### Optical filters and measurement parameters

Optical short pass or bandpass filters were added to the excitation light path and long pass filters to the emission light path to ensure clean excitation lines and to exclude scattered excitation radiation from the emission window, respectively (Table 1). The same excitation powers indicated in Table 1 were used throughout the study.

### Data collection and analysis

**Fig 2:**
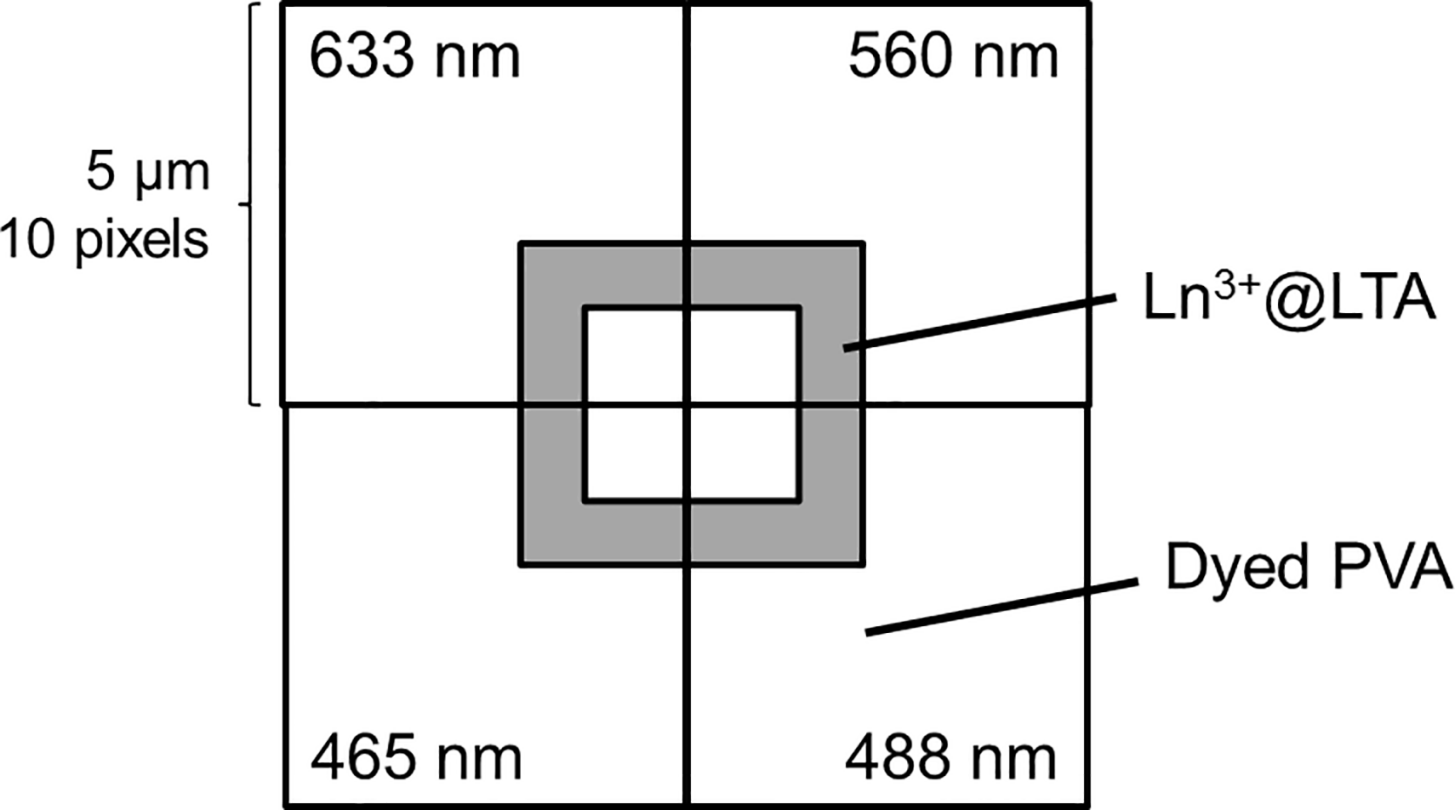
Areas used for imaging one zeolite using the four excitation wavelengths.

From the dyed polymer thin film a single zeolite was located for imaging. The zeolite and the dyed PVA-film surrounding it were imaged so that each of the four excitation wavelengths was used in separate corners of the zeolite by placing the center of the zeolite in one corner of the image (Fig 2). This minimized the bleaching of the dyes. An area of 5 μm×5 μm with 10 × 10 pixels was imaged with 1 s integration time per pixel, and the emission spectrum following the excitation at one of the four excitation wavelengths was recorded for each pixel. Then, another zeolite was located until both Eu(III)-doped and Tb(III)-doped zeolites were found. The images were created and analyzed with a home-written MATLAB (MathWorks, Natick, MA) routine. The pixels from the PVA thin film were analyzed for the fluorophore fluorescence and the pixels on top of the zeolites were analyzed for lanthanide luminescence, which could be identified by their uniquely narrow emission peaks from the broad spectral features of the fluorophores.

Most standard fluorescence microscopes do not have spectral imaging possibility, but instead, they use a set of optical filters to both select the excitation wavelength and the emission wavelength window. Therefore, to study contrast and crosstalk, we simulated the use of optical filters by integrating the spectra in the transmission wavelength region of commercial bandpass emission filters normally used for the selected three fluorophores. We simulated Edmund Optics emission filters for FITC, Texas Red and Cy5, and Chroma emission filters for FITC, mCherry and ATTO647N. FITC emission filters match the spectral characteristics of F18, Texas Red and mCherry emission filters match MitoTracker Red, and Cy5 emission filters match ATTO647N. The transmission wavelength region for the Edmund Optics filters were approximately 513–556 nm, 604–644 nm, and 672–712 nm, respectively, and for the Chroma laser bandpass filters 500–550 nm, 593–667 nm, and 669–741 nm, respectively. Because our microscopy setup contained a 532 nm long pass filter which becomes transparent around 539 nm, the emission window was started at 539 nm instead of 513 nm or 500 nm for the F18 channel. In our example the narrower emission window was compensated by a larger concentration of F18 dyes.

The fluorescent signal in the model system was achieved by subtracting the background from a thin-film sample containing only un-doped zeolites and no fluorophores. As we use scanning confocal microscopy to record spectral data in each pixel, we can then compare at least two separate pixels for both sample and background to perform standard deviation calculations: the average of two background pixels was separately subtracted from the signals of two pixels, and from these two signal intensity values the average and standard deviation were calculated.

## Results and discussion

### Model system

**Fig 3:**
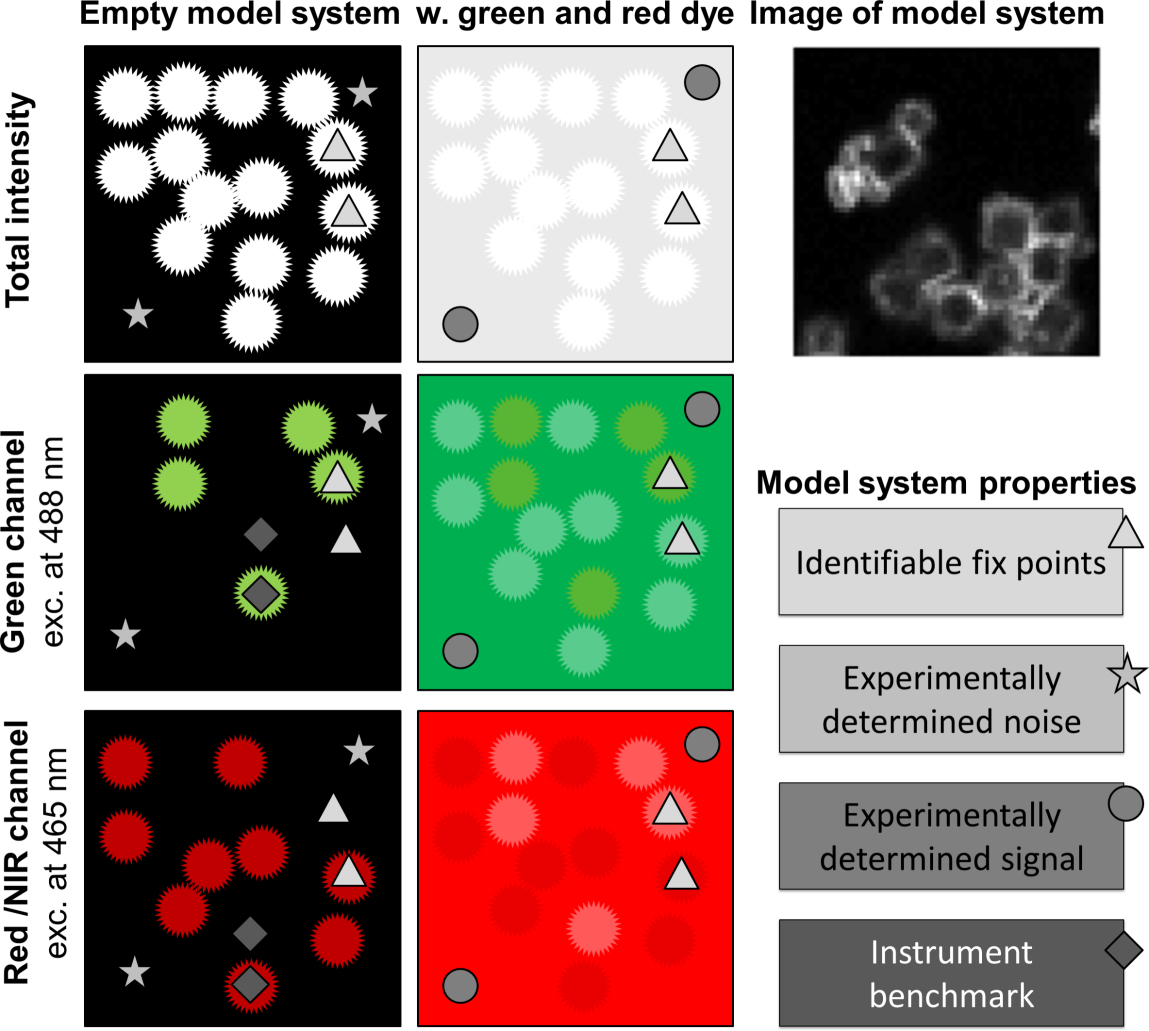
Schematic presentation of the model system. The model system is based on Ln(III)-doped zeolites and stained polyvinyl alcohol and is used to experimentally determine noise and fluorescent signals. Ln(III)-doped zeolites can be used as fix points for locating the region of interest and for instrument benchmarking.

We designed the model system to be readily available globally. It consists of Linde Type A zeolites doped with common salts of lanthanide(III) ions that are mixed in an aqueous solution of PVA and spin coated into thin films. Other emitters—with and without additional additives—can be dissolved in the PVA solution or embedded in the thin film. Using the model system, the fluorescence signals are recorded for each channel with the actual hardware and settings used for bioimaging, and the emitters are thereby benchmarked in actual measurement conditions. The model system allows the background signal of the sample to be experimentally determined, while the lanthanide centered emission from the zeolites acts as fix point and further may be used as an instrument benchmark, see Fig 3. As the specific signal of dyes and the background are determined by recording signals from samples with and without emitters, respectively, all fluorescence imaging methods and combinations of fluorophores can be evaluated directly. Furthermore, as the lanthanides can be excited with various laser lines, undergo two-photon excitation, have fluorescence lifetimes from nanoseconds to milliseconds, and emit across the entire visible and NIR range, most microscopy set-ups can be benchmarked using this model system.[27, 29–33] Here, we use spectral imaging to compare three fluorophores: F18, MitoTracker Red, and ATTO647N in a model system containing either terbium(III) or europium(III) doped zeolites (Fig 1).

Fluorescence microscope images recorded of the model system resemble the cartoon representation in Fig 3. The rationale behind this model system is to have a simple and robust sample that gives rise to a constant background and identifiable fluorescent signals following excitation with common laser lines and arc lamps. Many microscopes are fitted with a blue 488 nm laser, which is ideal for excitation of terbium(III) ions, while europium(III) ions can be used with a variety of excitation wavelengths in the range from 380–480 nm. The lanthanide ions are doped into zeolites that are readily imaged and differentiated from the background, see Fig 3. Zeolites were used as carriers for the lanthanides in our model system as the cation exchange is highly reproducible and gives rise to a system where the lanthanide ions are in a locked structure that generates highly reproducible emission spectra. Using the model system the background signal (stars, Fig 3) can be experimentally determined and compared to the achieved fluorescent signal (circle, Fig 3). The doped zeolites (triangles, Fig 3) can either be used just as fix points for locating the regions of interest, or the lanthanide centered emission may serve as an instrument benchmark (diamonds, Fig 3).

As the first example of using the model system to compare a set of dyes, we chose three dyes, each suited for channels (laser/filter-set) that are commonly used in fluorescence microscopy. The selected dyes were:

1. F18, a green membrane stain comprising a fluorescein with a C18 carbon chain,
2. MitoTracker Red, a red mitochondria stain essentially a rhodamine, and
3. ATTO647N, a far-red carborhodamine that can be conjugated, e.g., to nucleic acids for fluorescence *in situ* hybridization (FISH).

The photophysical properties of the dyes and lanthanide ions are compiled in Table 2. The selected dyes were dissolved at concentration of 150 μM (F18) and 0.1 μM (MitoTracker & ATTO647N) in 3 w/v% poly(vinyl alcohol) in water together with 20 mg/ml of Eu(III)-doped and 20 mg/ml of Tb(III)-doped zeolites. By spin coating the model system was assembled on a microscope slide (Fig 3). Samples were prepared without any dyes to record the background data, and in all possible combinations with one to three dyes.

**Table 2:** Photophysical properties of the chosen dyes and lanthanide ions.

	F18	MitoTracker Red	ATTO647N	Eu^3+^	Tb^3+^
QY	0.97^*a*^	0.43^*b*^	0.65^*c*^	0.052^*d*^	0.27 ^*d*^
ε_max_ / cm^-1^ (*λ / nm*)	92 300 (*500*)^*a*^	117 000 (*578*)^e^	150 000 (*646*)^*c*^	~2.8^f^ (*390*)	320^f^ (*220*)
ε(465 nm) / cm^-1^^*g*^	34 645	1 671	750	0.37^h^	
ε(488 nm) / cm^-1^^*g*^	71 371	3 882	900	0.035^h^	0.071^h^
ε(560 nm) / cm^-1^^*g*^		54 764	10 650		
ε(633 nm) / cm^-1^^*g*^			103 500		
Emission max / nm	517	598	664	616	544

^a^ from ref [34]

^*b*^ calculated (with QY = Brightness/ε) based on ref [23]

^*c*^ according to ATTO-TEC

^*d*^ based on our own QY-measurement of DOTA-complexes

^*e*^ according to the manufacturer

^*f*^according to absorption spectra by Carnall [35]

^*g*^ calculated from ThermoFisher Scientific SpectraViewer [36] as percentage from the ε_max_

^*h*^ based on our own absorption measurement of Eu and Tb acetates in water.

Images were recorded of all samples using excitation lines relevant for exciting the dyes (488 nm for F18 and Tb, 560 nm for MitoTracker Red, 633 nm for ATTO647N) and europium (465 nm). Here we used a home-built scanning fluorescence confocal microscope equipped with a white light laser source and a CCD-based spectrometer. For the exact settings please consult the methods section.

**Fig 4:**
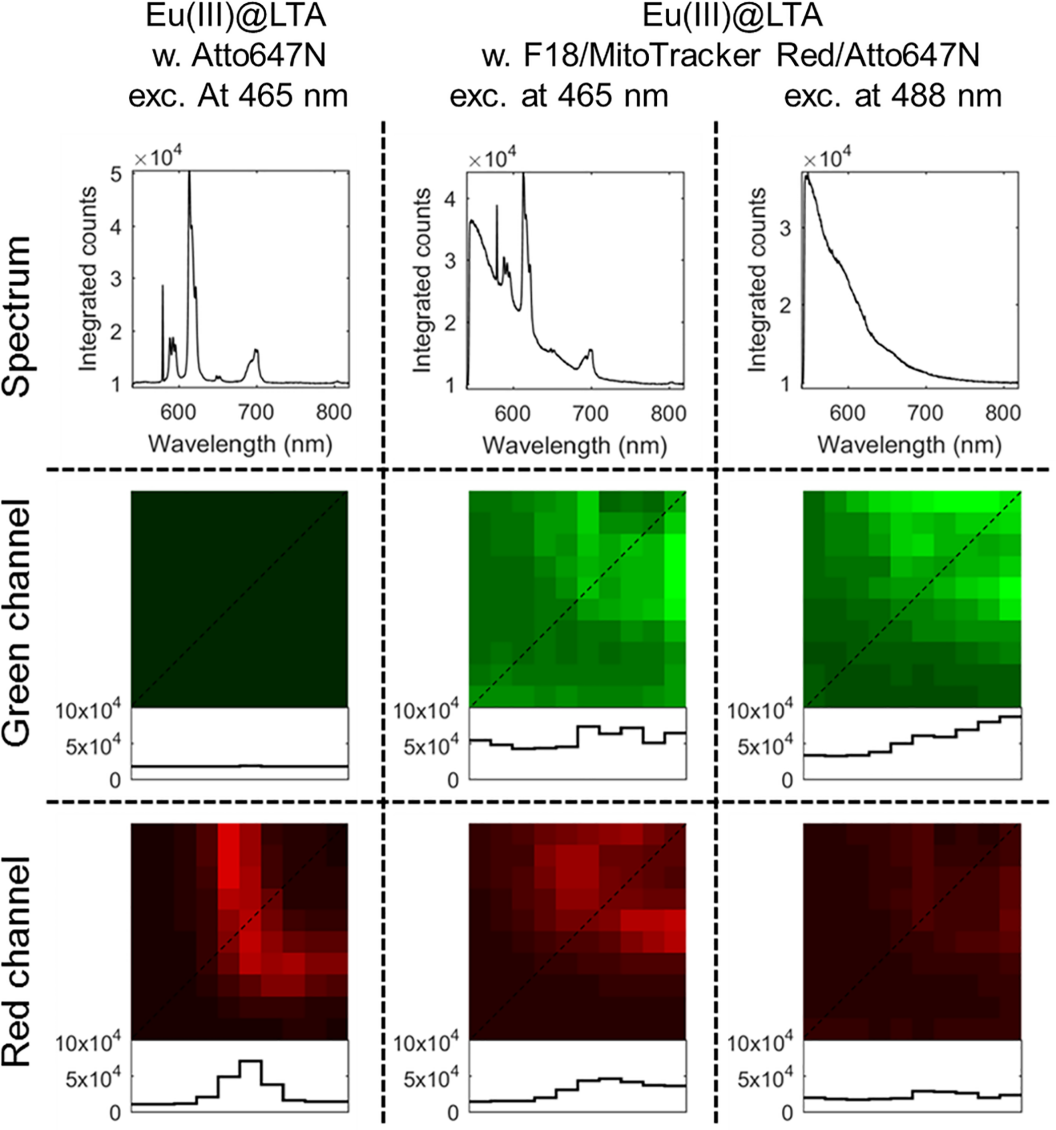
Model system for the scanning confocal fluorescence microscopy. Total intensity spectra and confocal fluorescence microscopy images of Eu(III)@LTA with ATTO467N in the PVA film following 465 nm excitation and Eu(III)@LTA with F18, MitoTracker Red, and ATTO467N in the PVA film following excitation at 465 nm or at 488 nm monitored through a green bandpass filter (539–556 nm) or a red bandpass filter (604–644 nm) of an Edmund Optics filter kit. The profiles of the diagonal lines (dotted) through the images are plotted beneath showing the pixel intensities.

Examples of the recorded images are shown in Fig 4. The total intensity spectrum of Eu(III)@LTA shows a typical emission spectrum of europium upon 465 nm excitation with sharp emission peaks. The confocal fluorescence microscopy image of Eu(III)@LTA through a red emission filter shows (see method section) a bright corner of a zeolite, in which the Eu(III)-dopants lighten up the edges while the center of the zeolite appears dark. The red emission window includes the sharp emission peak of Eu(III) at 615 nm, corresponding to ^5^D_0_ → ^7^F_2_ transition.[37] The plot profile shows a high contrast between the Eu(III)@LTA and the PVA surrounding. When looked through a green emission filter, only background signal is observed.

Similar images from the sample with Eu(III)@LTA in PVA dyed with F18 are also shown in Fig 4. Here upon 465 nm excitation, the total intensity spectrum clearly shows the uniquely narrow emission features of Eu(III) protruding from the broad emission peak of the F18, while upon 488 nm excitation only the F18 emission spectrum is observed. In the green channel images the fluorescence signal exclusively arises from F18 emission, while in the red channel image upon 465 nm excitation it is a mixture of Eu(III) emission and the tail of F18. Upon 488 nm excitation no Eu(III) emission is observed, but still some signal is observed in the red channel originating from the broad emission of F18. So, while we can readily identify the emitting species in spectrally resolved imaging, the model system shows that in a filter based experiment we would record the fluorescent signal corresponding to F18 emission in the red channel too. No signal is detected from this sample when using the traditional laser line for the red channel (here 560 nm was used, images not shown). Images similar to those shown in Fig 4 have been analyzed in the following sections for all three dyes upon all four excitation wavelengths and three detection channels.

The model system was made by spin coating dilute samples on coverslips. This may not be ideal as the films should be significantly thicker than the focal depth of the microscope used. We are currently working on developing a simple procedure to make thicker films and on testing these in different microscopes.

### The fluorescent signals

The parameter that determines the outcome of an imaging experiment is the fluorescent signal. The model system is set up to determine the fluorescent signal, the background signal and the contrast of any given set-up. Here, we evaluated the fluorescent signal and compared the measured signal to the brightness of the probe, and we compared the fluorescent signal achieved through emulating two different filter kits.

**Fig 5:**
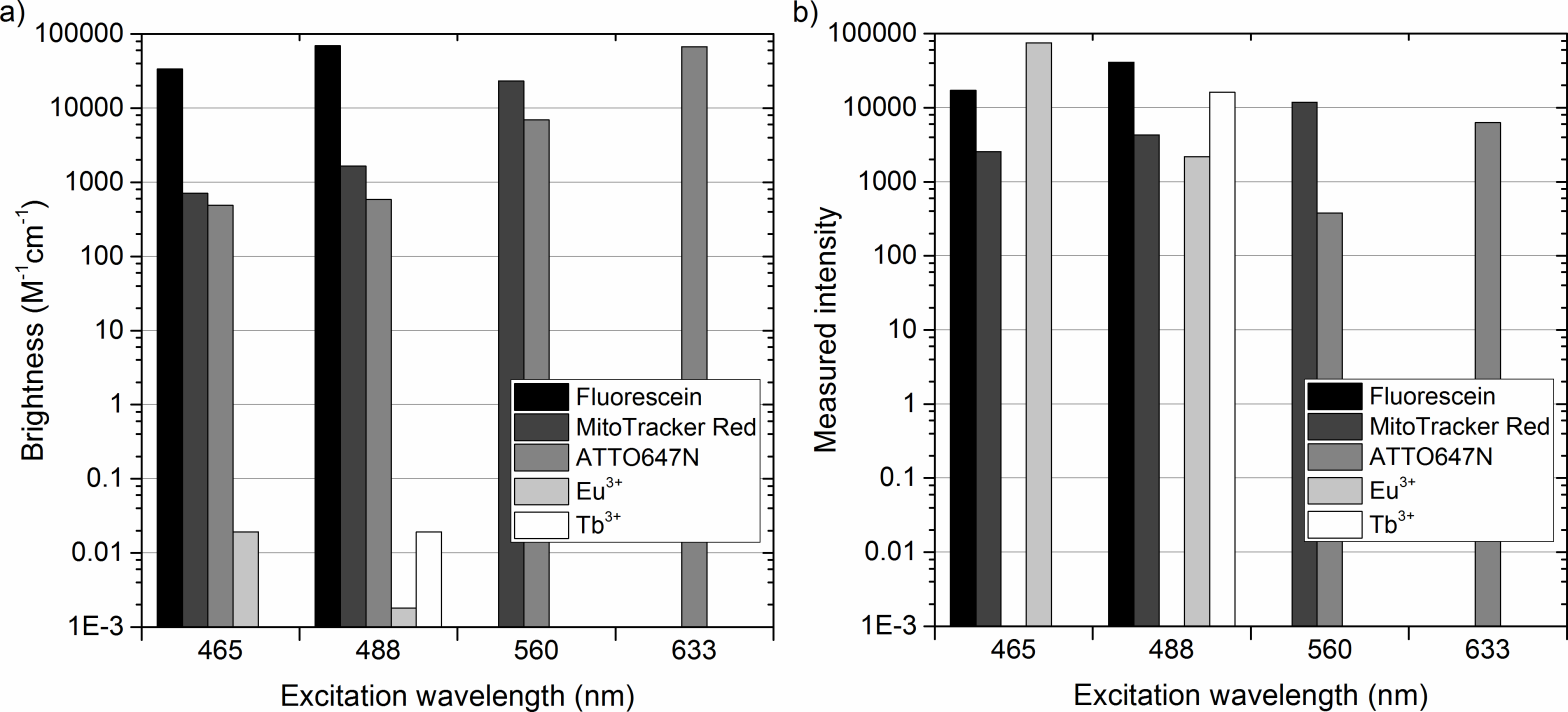
The brightness and measured intensity of the dyes in the specific conditions used. **a)** Brightness is the product of the quantum yield and the molar absorptivity of the dye. The brightnesses of Eu(III) and Tb(III) are calculated from the quantum yield of their DOTA-complexes and the absorptivities of their acetate salts (our own measurements). **b)** The measured intensities correspond to the fluorescent signals arising from each fluorescent probe (fully integrated spectra) upon different excitation wavelengths.

The theoretical brightness of each dye was calculated by multiplying the quantum yield of the dyes with their molar absorptivity at actual wavelengths used for excitation (Fig 5A). F18 and ATTO647N have a high brightness of ~70 000 M^-1^cm^-1^ upon 488 nm and 633 nm excitation, respectively, while the brightness of MitoTracker Red is ~23 000 M^-1^cm^-1^ upon 560 nm excitation. The calculated brightness of lanthanide ions is very poor due to their low molar absorptivity, which is the result of the forbidden nature of *f*-*f* transitions within the 4*f* manifold.[37] As limited information is available in the literature on direct excitation of lanthanide ions, the brightness was calculated from the quantum yield of DOTA-complexes of Eu(III) and Tb(III), while the molar absorptivities in the calculations are based on absorption measurement of Eu(III) and Tb(III) acetates (Fig 5A, Table 2). The brightness values given for the lanthanide(III) ions are probably over-estimations for the actual conditions inside zeolites, but give the “best case scenarios”. The excitation at 465 nm promotes the ^7^F_0_ → ^5^D_2_ transition in Eu(III), while 488 nm radiation promotes the ^7^F_6_ → ^5^D_4_ transition in Tb(III). The molar absorptivity of both of these transitions is less than 0.1 M^-1^cm^-1^ leading to a brightness of 0.02 M^-1^cm^-1^. Fig 5B shows the actual measured intensities of the dyes and lanthanide(III) ions using the model system and our microscope setup, calculated by fully integrating their emission spectra upon different excitation wavelengths.

Cursory inspection of Fig 5 shows a rather poor correlation between the brightness and the measured intensity. It should be noted, that these values depend on many parameters, among others, the dye concentration, excitation power, detector efficiency, transmission of filters and dichroic mirrors, bleaching rate and integration time. If we use the measured intensities of F18 and MitoTracker Red as a benchmark, ATTO647N is nearly an order of magnitude less intense than what is predicted by the brightness. Note the molarity is 1500 times higher for F18 than for MitoTracker Red and ATTO647N, the two latter having identical dye loading at 0.1 μM. In our system, F18 underperforms dramatically, while MitoTracker Red and ATTO647N behave more as expected. Most surprising is the measured intensity of Eu(III) and Tb(III) upon direct excitation at 465 nm and 488 nm, respectively. The measured emission is the same order of magnitude with the organic dyes despite their low molar absorptivity and turnover rate (Fig 5B). A direct comparison of Eu(III) and F18 highlights this fact: The brightness of Eu(III) following direct excitation is <<1, while that of F18 is ≈100.000. Furthermore, the excited state lifetime of europium is ≈500.000 ns, while that of F18 is ≈ 5 ns. This difference implies that at similar concentration the F18 stained areas should be able to emit >100.000.000.000 times more photons per second than Eu(III) stained areas. Even considering the difference in dye loading that favors Eu(III) by an estimated factor of ~300, the fluorescent signal from F18 should be significantly larger than that of Eu(III). The lower performance of F18 is influenced by its faster bleaching rate, and other effects like pH stability will also play a role. The result illustrates that even low brightness dyes can give a good contrast under specific experimental and sample preparation conditions, illustrating the need for comparison in a benchmark on unequal terms/actual experimental conditions.

**Fig 6:**
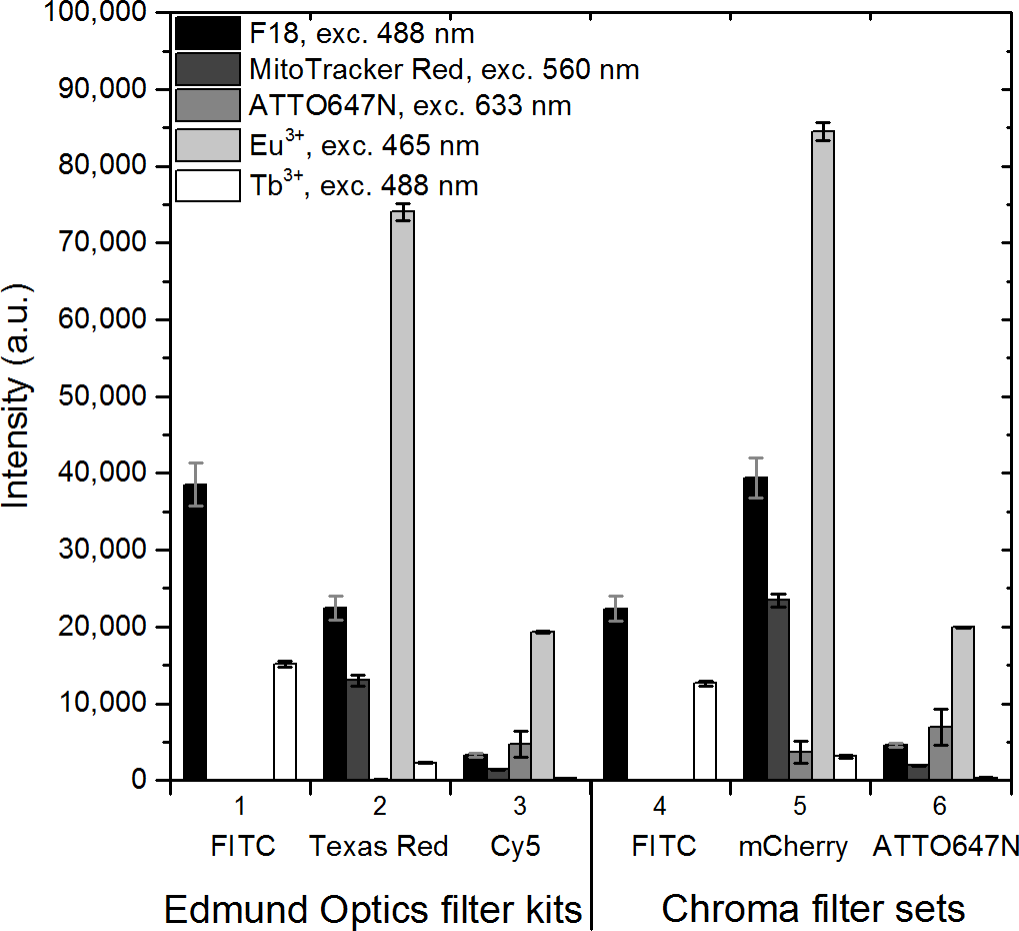
Fluorescent signal of each dye at their optimal excitation wavelength. Each emission window is simulated by integrating the acquired emission spectra only in the wavelength area determined by the commercial optical emission bandpass filters. The Edmund Optics filter kits are a standard set of filters for 1) FITC, 2) Texas Red, and 3) Cy5, with wavelength ranges of the emission bandpass filters at 539–556 nm, 604–644 nm, and 672–712 nm, respectively. The Chroma laser bandpass filter sets for 4) FITC, 5) mCherry, and 6) ATTO647N had wavelength ranges of 539–550 nm, 593–667 nm, and 669–741 nm, respectively. The background from PVA-sample without dyes was subtracted. Error bars illustrate the standard deviation in the observed signals of two separate pixels in the images.

Next, we calculated the fluorescent signal of the dyes by subtracting the background signal using data from a model system with no dyes (as illustrated in Fig 3). We sectioned the measured spectra into three emission windows, which simulate the use of optical emission filters of commercially available filter sets. The fluorescent signal was determined for the dyes at their optimal excitation wavelengths (Fig 6). The fluorescent signal of Tb(III) was high using filter 1 and filter 4, while Eu(III) exhibited a high signal using filter 2 and filter 5. Closer inspection of Fig 6 shows that the wide emission spectrum of F18 gives rise to a significant fluorescent signal in the emission window designated to MitoTracker Red (filters 2 and 5). In fact, with the staining conditions used the signal from high concentration of F18 in the red channel following 488 nm is higher than that of MitoTracker Red following 560 nm excitation (Fig 6). Therefore, we decided to use our model system to investigate the possible crosstalk between channels.

### Crosstalk investigation

When using multiple fluorophores with broad spectral features simultaneously to stain a sample, some crosstalk between them is inevitable. This can particularly cause problems in co-localization, Förster resonance energy transfer (FRET) and multilabeling studies. Spectral crosstalk includes both excitation crosstalk (cross-excitation), where multiple fluorophores are excited with a single excitation wavelength, and emission bleed-through, where unwanted fluorescent signal from a dye with overlapping emission (and excitation) spectrum is detected in the emission window. Crosstalk, as well as differences in the brightness or concentrations of the dyes, leads to specificity problems, where the origin of the detected photon is unclear. Several solutions have been proposed to deal with cross-talk.[38–45] As the model system gives direct access to data from systems with and without co-localization, the amount of crosstalk can be readily determined.

**Fig 7:**
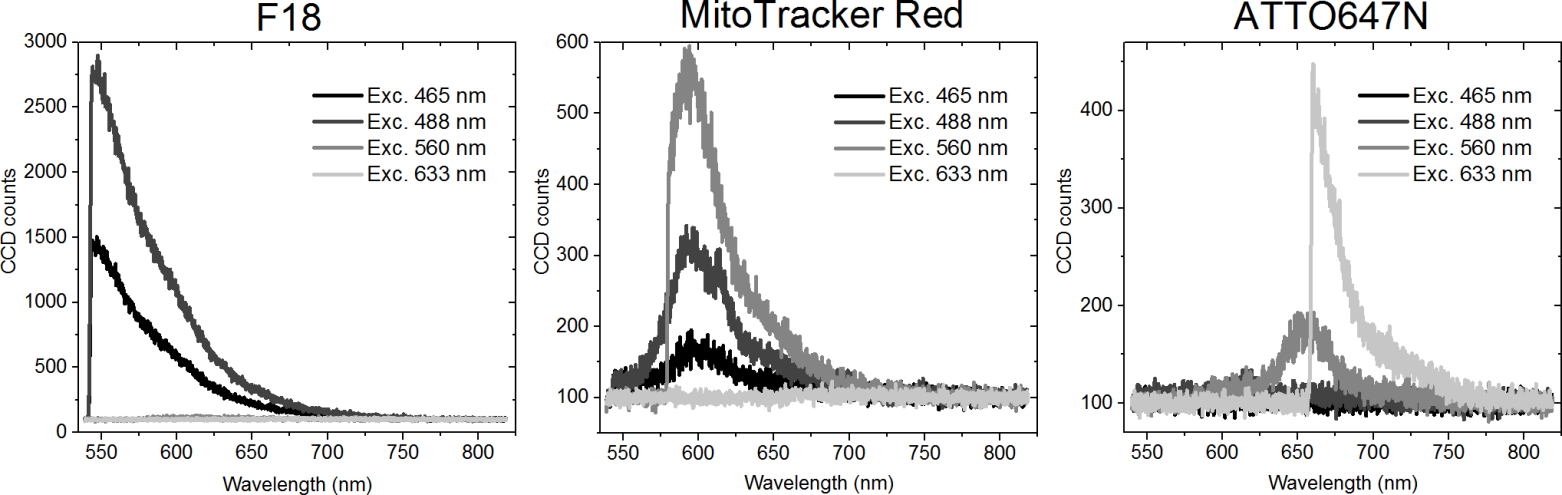
Cross-excitation of the dyes. Emission spectra of F18, MitoTracker Red, and ATTO647N upon excitation at 465 nm, 488 nm, 560 nm, and 633 nm.

Here, we can take a step further by exploiting the fact that we record the spectra of each dye. By recording the spectra following excitation using the four laser lines with wavelengths 465 nm, 488 nm, 560 nm and 633 nm, the excitation crosstalk can be demonstrated (Fig 7). Fig 7 shows the spectra of individual fluorophores upon the different excitation wavelengths. Naturally, no emission is observed when an excitation wavelength beyond the red side of the excitation maximum is used. Excitation crosstalk is the most pronounced for MitoTracker Red, while a small amount of excitation of ATTO647N can be seen using the 560 nm laser line.

**Fig 8:**
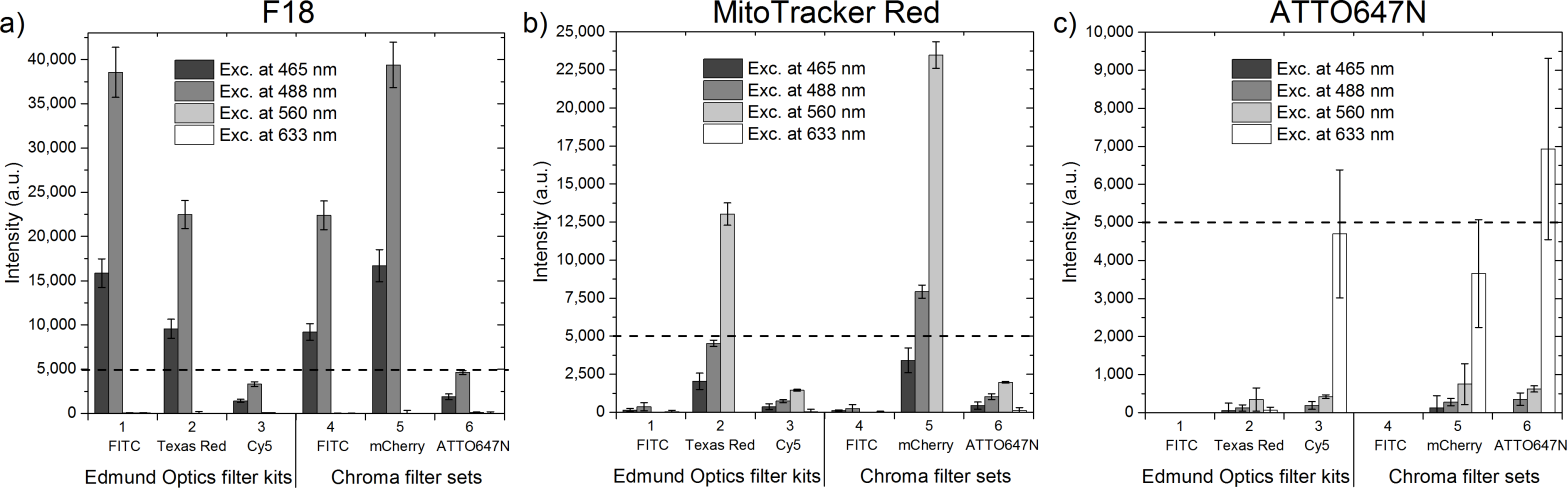
Fluorescent signal of dyes in simulated emission windows upon different excitation wavelengths. Each emission window is imitated by integrating the acquired emission spectra only in the wavelength area determined by the commercial optical bandpass filters. The Edmund Optics filter kits are a standard set of filters for 1) FITC, 2) Texas Red, and 3) Cy5, with transmission wavelength ranges of the emission bandpass filters at 539–556 nm, 604–644 nm, and 672–712 nm, respectively. The Chroma laser bandpass filter sets for 4) FITC, 5) mCherry, and 6) ATTO647N had wavelength ranges of 539–550 nm, 593–667 nm, and 669–741 nm, respectively. The background from PVA-sample without dyes was subtracted. The dashed lines are guides for the eye at intensity of 5000 a.u. Error bars represent the standard deviation of two separate pixels in each image.

The crosstalk originating from emission bleed-through was determined by emulating the commercial filter sets commonly used in fluorescence microscopy to separate the signals of the most common fluorophore combinations. The measured fluorescent signal following excitation at 465 nm, 488 nm, 560 nm, and 633 nm is plotted for each filter in Fig 8. The dashed line is a guide for the eye to better illustrate the intensity differences of the three dyes. It is evident that F18 emission can be observed in all emission windows upon excitation at 465 nm or 488 nm. Its fluorescence signal (excitation at 488 nm) exceeds that of MitoTracker Red upon 560 nm excitation in the filter channels 2 and 5. Only the emission intensity of the ATTO647N dye upon 633 nm excitation is higher than the F18 signal in the filter channels 3 and 6. This clearly demonstrates the problem of different relative concentrations of dyes in samples: the emission of F18 overshadows the emission of the two other dyes and would give rise to false positives in co-localization experiments.

### Bleaching

**Fig 9:**
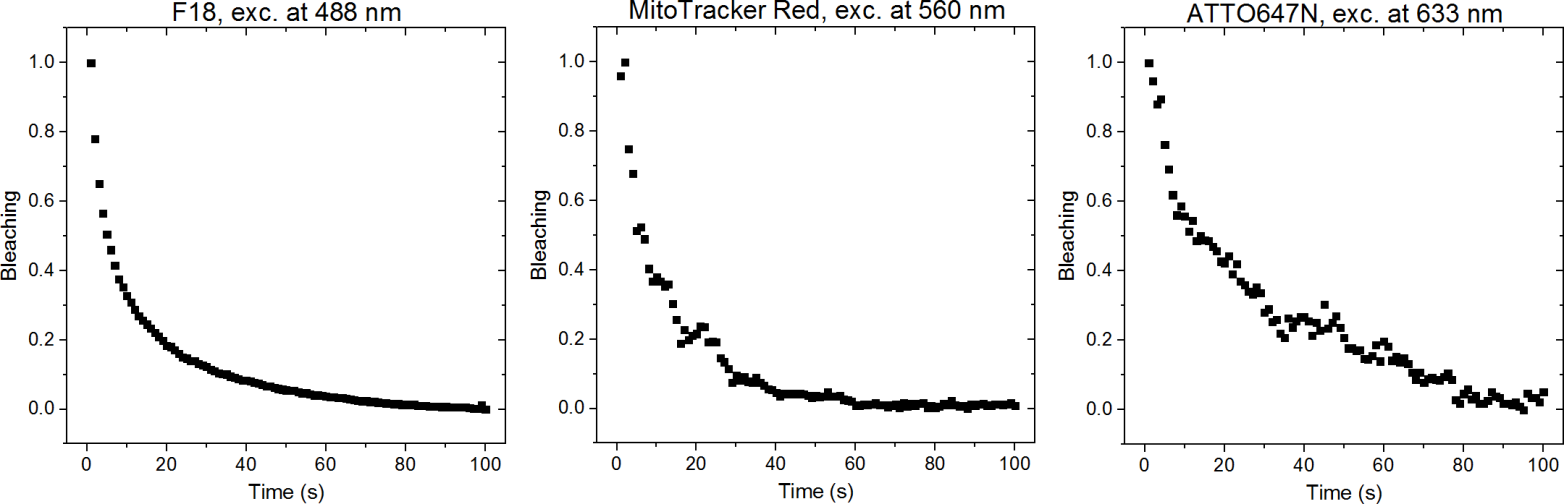
Bleaching of F18, MitoTracker Red and ATTO647N. The spectra were recorded in 1 s intervals for 100 s. For F18 the whole spectrum was integrated, for MitoTracker Red only the wavelength range of 578–817 nm was integrated, and for ATTO647N the wavelength range of 567–817 nm was integrated. The integrated intensities were normalized to the range of 0–1. The excitation powers can be found in the methods section.

The model system is flexible as most water soluble compounds can be included in the PVA film. This allows the study of the effects of additives and multiple dyes on different important parameters. As the actual experimental parameters are used when imaging the model system, aspects such as bleaching are readily addressed. In our example we studied the bleaching of the dyes by measuring the emission spectrum from a single pixel in 1 s intervals for a total of 100 s. Fig 9 shows the bleaching of F18 dye upon excitation at 488 nm, MitoTracker Red upon 560 nm, and ATTO647N upon 633 nm excitation. As expected [20], F18 bleaches very fast losing about 70% of the intensity during the first 10 seconds and then levels out at a level corresponding to the background signal. Surprisingly, neither MitoTracker Red nor ATTO647N performed much better. Both dyes have a fast 5–10 second component where about 50% of the intensity is lost. MitoTracker Red has a long component where all signal is lost after 40 seconds, while ATTO647N has long lived population that survives longer before all signal is lost at 100 seconds. In the model system bleaching is readily evaluated using single pixel accelerated bleaching, as done in Fig 9, or by repeated imaging in one or more channels in order to investigate the effect of multiple exposures of various laser lines on each of the fluorophores used.

## Conclusion

We have proposed a model system that can be used as a common benchmark for experimentally investigating the choice of probe, sample preparation methods, microscopes, imaging techniques, and data processing in fluorescent bioimaging. We have demonstrated the model system by comparing a green, red and far-red fluorescent probe. Using a specific set of imaging parameters, we have evaluated bleaching, the magnitude of the fluorescent signal, and crosstalk in samples stained with the three selected dyes. In the model system the background signal and fluorescent signals from the individual dyes are readily experimentally determined using the exact same parameters that are used in a bioimaging experiment.

Our aim is to enable direct comparison of image quality despite large differences in sample preparation routines, imaging technique, hardware set-ups, image analysis, dye loading etc. By reporting images of a model system the quality of experimental data and imaging methods can be directly seen. The model system has the actual dye concentrations, has been imaged using the actual parameters and the images of the model system must be created using the same image analysis protocol as the biologically relevant samples. Images of the biological samples may be difficult to obtain, but the model system is fully reproducible and can serve as a benchmark in fluorescence based bioimaging.

In the example presented here, we found that the brightness of a molecular probe is not necessarily the only factor that determines the achieved fluorescent signal. Further we found that crosstalk between channels must be considered in samples that are going to be excited with multiple wavelengths or when emission needs to be collected in adjacent spectral regions. Finally we conclude that the example clearly demonstrates that molecular probes must be compared in a model system using the actual imaging conditions.

## Supporting information

S1 File Supporting information. This document describes the full lists of files included in S2 File.zip and S3 File.zip as well as the experimental details.
(PDF)

S2 File Image files. (ZIP)

S3 File Spectral data files. (ZIP)
