# Supplementary material for: Investigating dye performance and crosstalk in fluorescence enabled bioimaging using a model system
Source: PLoS One. 2017 Nov 27;12(11):e0188359. doi: 10.1371/journal.pone.0188359 (PMC5703511; doi:10.1371/journal.pone.0188359)
Supplement: S1 File — This document describes the full lists of files included in S2 File.zip and S3 File.zip as well as the experimental details. (PDF) [file pone.0188359.s001.pdf]

Supporting information

Investigating dye performance and crosstalk in fluorescence enabled bioimaging using a model system

Riikka Arppe,[1] Miguel R. Carro-Temboury,[1] Casper Hempel,[2*] Tom Vosch[1*] and Thomas Just Sørensen[1*]

[1]Nano-Science Center & Department of Chemistry, University of Copenhagen, Copenhagen, Denmark

[2] Dept of Micro- and Nanotechnology, Technical University of Denmark, Kgs Lyngby, Denmark

Table of contents

Table of contents 1
Content of the SI:..2
Samples: 2
Imaging the samples:..2
Image files in S2_file.zip:..3
Spectral data in S3_file.zip:..4

Content of the SI:

The SI contains this document, files with image data, and files with spectral data. All data are from the seven samples in the list below. Additional data has been added per request of the reviewers; this data is added at the end of this document.

Samples:

S1: 100 µg/ml F18, Tb-zeolites, Eu-zeolites; in 3% (w/v) PVA

S2: 0.1 µM MitoTracker Red, Tb-zeolites, Eu-zeolites; in 3% (w/v) PVA

S3: 0.1 µM ATTO647N, Tb-zeolites, Eu-zeolites, EuTb-zeolites; in 3% (w/v) PVA

S4: 100 µg/ml F18, 0.1 µM MitoTracker Red, Tb-zeolites, Eu-zeolites; in 3% (w/v) PVA

S5: 0.1 µM MitoTracker Red, 0.1 µM ATTO647N, Tb-zeolites, Eu-zeolites; in 3% (w/v) PVA

S6: 100 µg/ml F18, 0.1 µM ATTO647N, Tb-zeolites, Eu-zeolites; in 3% (w/v) PVA

S7: 100 µg/ml F18, 0.1 µM MitoTracker Red, 0.1 µM ATTO647N, Tb-zeolites, Eu-zeolites; in 3% (w/v) PVA

Imaging the samples:

A single zeolite was located and it surroundings were imaged using the four excitation wavelengths (465 nm, 488 nm, 560 nm and 633 nm) so that each excitation wavelength was used in separate corners of the zeolite (Figure S1). This was done in order to minimize the bleaching of the dyes.

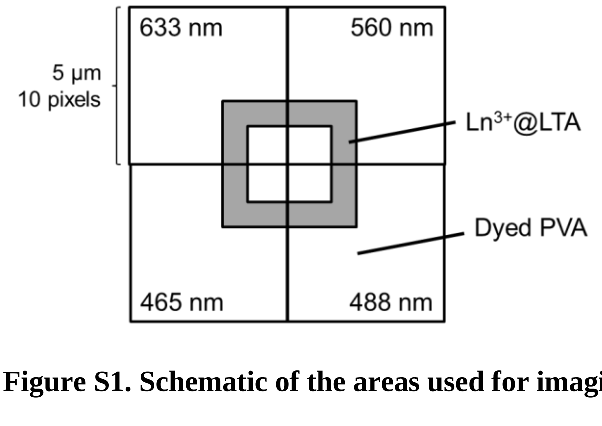

Figure S1. Schematic of the areas used for imaging one zeolite using the four excitation wavelengths.

Image files in S2_file.zip:

The file names show the sample number, the lanthanide dopant, the excitation wavelength, and the location of the image in respect to the zeolite in the middle, respectively (see Figure S1).

S1:
- S1_Tb_465_lowleft
- S1_Tb_488_lowright
- S1_Tb_560_topright
- S1_Tb_633_topleft
- S1_Eu_465_lowleft
- S1_Eu_488_lowright
- S1_Eu_560_topright
- S1_Eu_633_topleft

S2:
- S2_Tb_465_lowleft
- S2_Tb_488_lowright
- S2_Tb_560_topright
- S2_Tb_633_topleft
- S2_Eu_465_lowleft
- S2_Eu_488_lowright
- S2_Eu_560_topright
- S2_Eu_633_topleft

S3:
- S3_EuTb_560_topright
- S3_EuTb_633_topleft
- S3_Tb_465_lowleft
- S3_Tb_488_lowright
- S3_Tb_560_topright
- S3_Tb_633_topleft
- S3_Eu_465_lowleft
- S3_Eu_488_lowright
- S3_Eu_560_topright
- S3_Eu_633_topleft

S4:
- S4_Tb_465_lowleft
- S4_Tb_488_lowright
- S4_Tb_560_topright
- S4_Tb_633_topleft
- S4_Eu_465_lowleft
- S4_Eu_488_lowright
- S4_Eu_560_topright
- S4_Eu_633_topleft

S5:
- S5_Tb_465_lowleft
- S5_Tb_488_lowright
- S5_Tb_560_topright
- S5_Tb_633_topleft
- S5_Eu_465_lowleft
- S5_Eu_488_lowright
- S5_Eu_560_topright
- S5_Eu_633_topleft

S6:
- S6_Tb_465_lowleft
- S6_Tb_488_lowright
- S6_Tb_560_topright
- S6_Tb_633_topleft
- S6_Eu_465_lowleft
- S6_Eu_488_lowright
- S6_Eu_560_topright
- S6_Eu_633_topleft

S7:
- S7_Tb_465_lowleft
- S7_Tb_488_lowleft
- S7_Tb_560_topright
- S7_Tb_633_topleft
- S7_Eu_465_lowleft
- S7_Eu_488_lowright
- S7_Eu_560_topright
- S7_Eu_633_topleft

Empty zeolites_PVA no dyes:
- EmptyZ_PVA no dyes_465_lowleft
- EmptyZ_PVA no dyes_488_lowright
- EmptyZ_PVA no dyes_560_topright
- EmptyZ_PVA no dyes_633_topleft

Spectral data in S3_file.zip:

Spectra were acquired from both the PVA-background and from the brightest pixel on top of the zeolite. The file names indicate the sample number, the lanthanide dopant, the excitation wavelength, and the pixel in the corresponding image.

S1_PVA:

- S1_Tb_465_x1y2
- S1_Tb_488_x10y1
- S1_Tb_560_x10y10
- S1_Tb_633_x1y2

S1_Zeolite:

- S1_Tb_465_x4y10
- S1_Tb_488_x7y10
- S1_Tb_560_x7y1
- S1_Tb_633_x8y4
- S1_Eu_465_x5y10
- S1_Eu_488_x9y10
- S1_Eu_560_x4y3
- S1_Eu_633_x10y8

S2_PVA:

- S2_Eu_465_x10y1
- S2_Eu_488_x10y10
- S2_Eu_560_x9y10
- S2_Eu_633_x1y10

S2_Zeolite:

- S2_Tb_465_x7y8
- S2_Tb_488_x7y8
- S2_Tb_560_x5y6
- S2_Tb_633_x5y4
- S2_Eu_465_x10y9
- S2_Eu_488_x5y10
- S2_Eu_560_x3y4
- S2_Eu_633_x10y5

S3_PVA:

- S3_Eu_465_x1y1
- S3_Eu_488_x8y2
- S3_EuTb_560_x5y10
- S3_EuTb_633_x1y10

S3_Zeolite:

- S3_Tb_465_x6y10
- S3_Tb_488_x1y10
- S3_Tb_560_x2y4
- S3_Tb_633_x9y4
- S3_Eu_465_x5y9
- S3_Eu_488_x3y5
- S3_Eu_560_x2y8
- S3_Eu_633_x8y5

S4_PVA:

- S4_Tb_465_x1y1
- S4_Tb_488_x10y1
- S4_Tb_560_x8y10
- S4_Tb_633_x10y10

S4_Zeolite:

- S4_Tb_465_x5y10
- S4_Tb_488_x6y10
- S4_Tb_560_x4y4
- S4_Tb_633_x7y3
- S4_Eu_465_x5y10
- S4_Eu_488_x1y9
- S4_Eu_560_x1y6
- S4_Eu_633_x7y3

S5_PVA:

- S5_Tb_465_x10y1
- S5_Tb_488_x10y1
- S5_Tb_560_x10y10
- S5_Tb_633_x1y10

S5_Zeolite:

- S5_Tb_465_x6y7
- S5_Tb_488_x1y5
- S5_Tb_560_x1y5
- S5_Tb_633_x10y5
- S5_Eu_465_x4y7
- S5_Eu_488_x1y6

- S5_Eu_560_x1y4
- S5Eu_633_x10y5

S6_PVA:
- S6_Eu_465_x1y10
- S6_Eu_488_x10y10
- S6_Eu_560_x10y10
- S6_Eu_633_x1y10

S6_Zeolite:
- S6_Tb_465_x7y10
- S6_Tb_488_x8y10
- S6_Tb_560_x3y5
- S6_Tb_633_x7y1
- S6_Eu_465_x7y10
- S6_Eu_488_x4y10
- S6_Eu_560_x2y3
- S6_Eu_633_x10y3

S7_PVA:
- S7_Tb_465_x1y1
- S7_Tb_488_x10y1
- S7_Eu_560_x1y10
- S7_Eu_633_x1y10

S7_Zeolite:
- S7_Tb_465_x10y7
- S7_Tb_488_x10y7
- S7_Tb_560_x1y2
- S7_Tb_633_x8y4
- S7_Eu_465_x8y10
- S7_Eu_488_x4y10
- S7_Eu_560_x1y6
- S7_Eu_633_x10y10

PVA without dyes:
- 465_x1y2
- 488_x10y1
- 560_x7y10
- 633_x1y10

Empty zeolites:
- 465_x8y10
- 488_x5y10
- 560_x4y4
- 633_x7y3
