# Supplementary figures and images for: Investigating dye performance and crosstalk in fluorescence enabled bioimaging using a model system
Source: PLoS One. 2017 Nov 27;12(11):e0188359. doi: 10.1371/journal.pone.0188359 (PMC5703511; doi:10.1371/journal.pone.0188359)

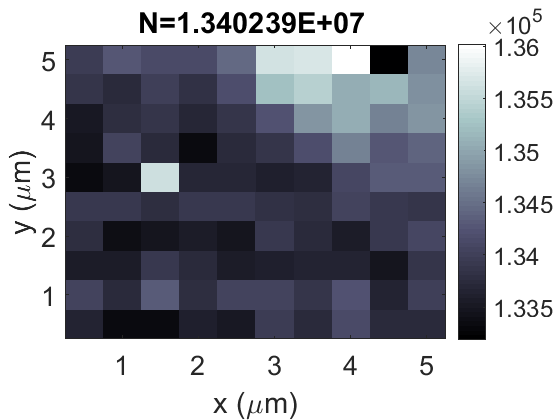

Supplement: S2 File — (ZIP) [file pone.0188359.s002.zip › Images/EmptyZ_PVA no dyes_465_lowleft.tif]

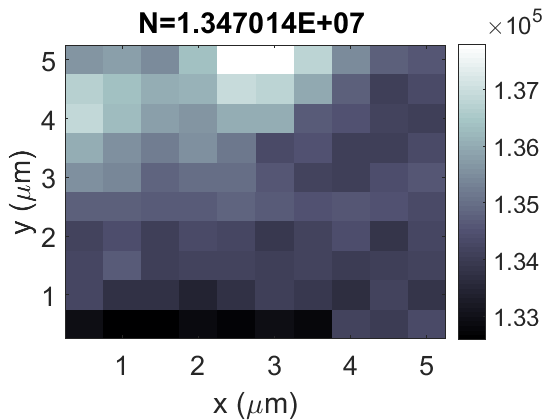

Supplement: S2 File — (ZIP) [file pone.0188359.s002.zip › Images/EmptyZ_PVA no dyes_488_lowright.tif]

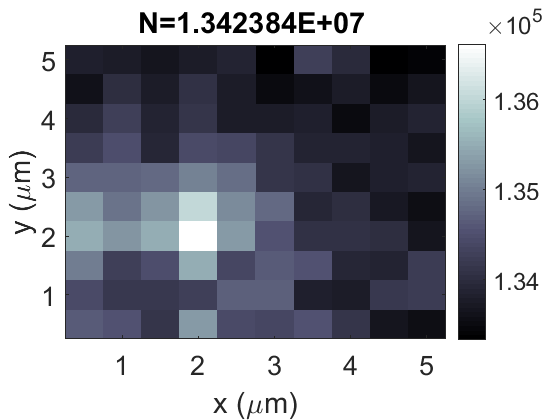

Supplement: S2 File — (ZIP) [file pone.0188359.s002.zip › Images/EmptyZ_PVA no dyes_560_topright.tif]

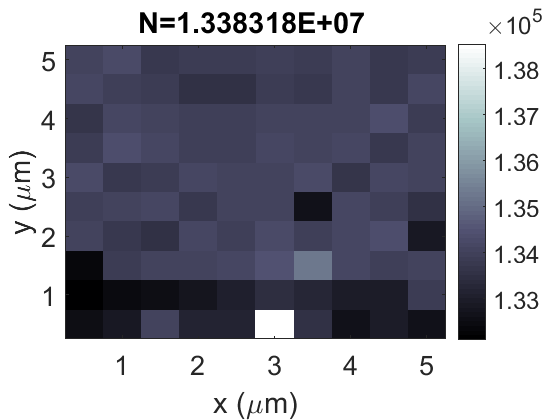

Supplement: S2 File — (ZIP) [file pone.0188359.s002.zip › Images/EmptyZ_PVA no dyes_633_topleft.tif]

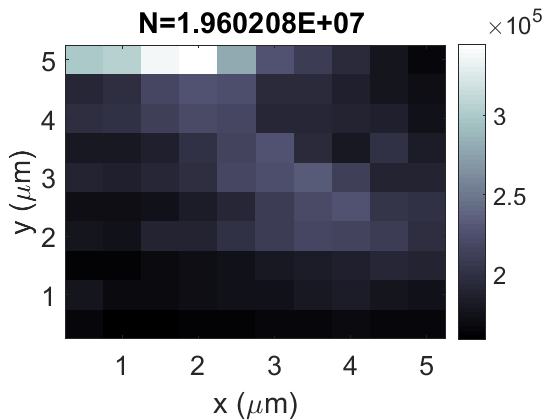

Supplement: S2 File — (ZIP) [file pone.0188359.s002.zip › Images/S1_Eu_465_lowleft.tif]

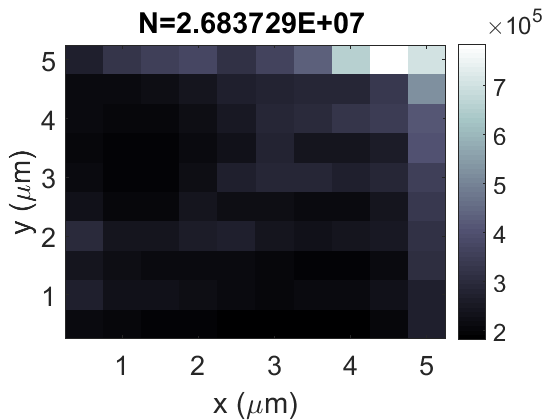

Supplement: S2 File — (ZIP) [file pone.0188359.s002.zip › Images/S1_Eu_488_lowright.tif]

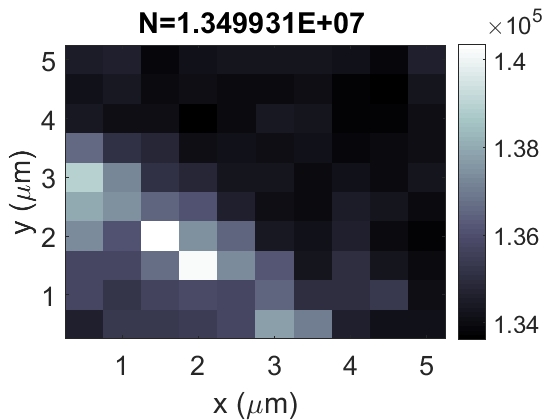

Supplement: S2 File — (ZIP) [file pone.0188359.s002.zip › Images/S1_Eu_560_topright.tif]

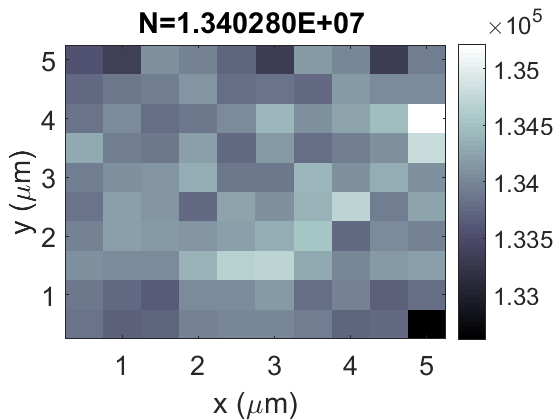

Supplement: S2 File — (ZIP) [file pone.0188359.s002.zip › Images/S1_Eu_633_topleft.tif]

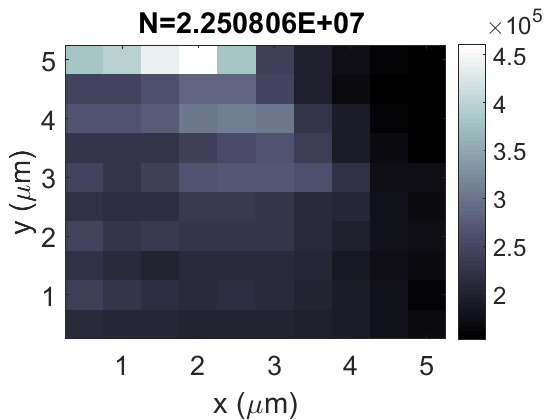

Supplement: S2 File — (ZIP) [file pone.0188359.s002.zip › Images/S1_Tb_465_lowleft.tif]

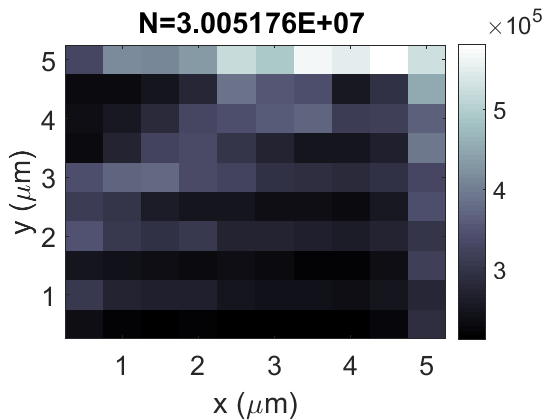

Supplement: S2 File — (ZIP) [file pone.0188359.s002.zip › Images/S1_Tb_488_lowright.tif]

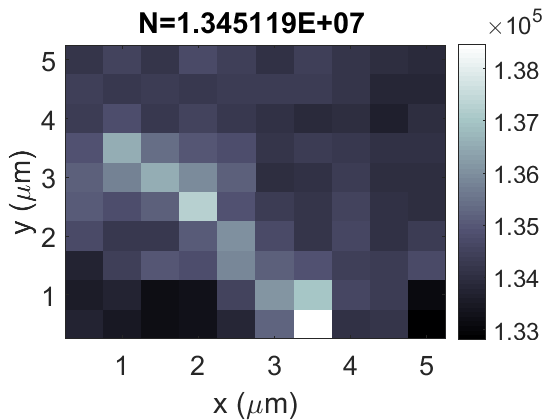

Supplement: S2 File — (ZIP) [file pone.0188359.s002.zip › Images/S1_Tb_560_topright.tif]

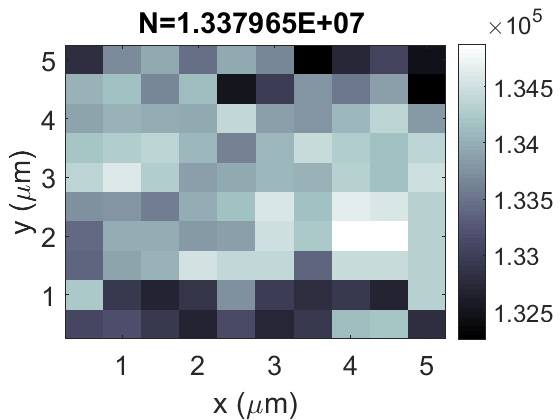

Supplement: S2 File — (ZIP) [file pone.0188359.s002.zip › Images/S1_Tb_633_topleft.tif]

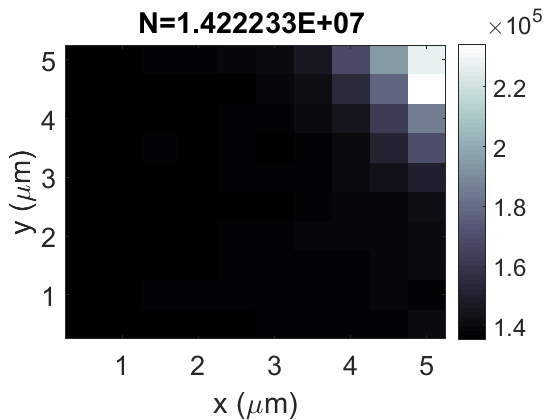

Supplement: S2 File — (ZIP) [file pone.0188359.s002.zip › Images/S2_Eu_465_lowleft.tif]

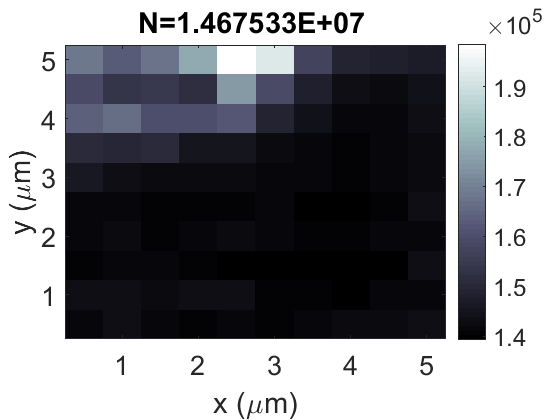

Supplement: S2 File — (ZIP) [file pone.0188359.s002.zip › Images/S2_Eu_488_lowright.tif]

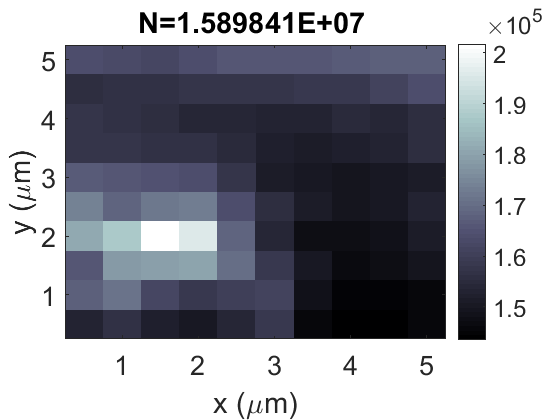

Supplement: S2 File — (ZIP) [file pone.0188359.s002.zip › Images/S2_Eu_560_topright.tif]

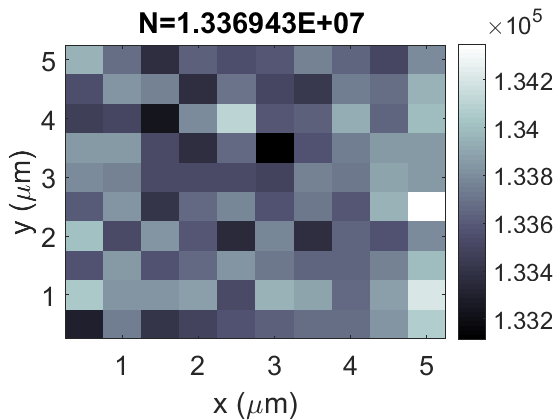

Supplement: S2 File — (ZIP) [file pone.0188359.s002.zip › Images/S2_Eu_633_topleft.tif]

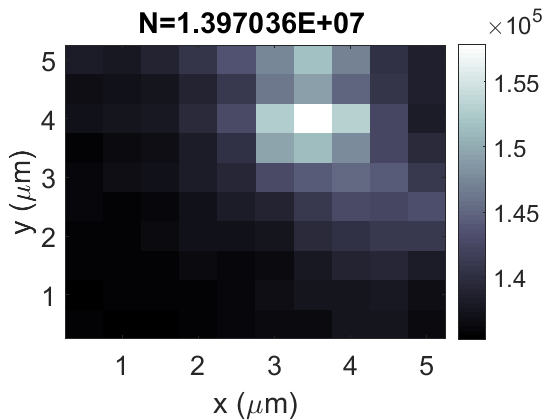

Supplement: S2 File — (ZIP) [file pone.0188359.s002.zip › Images/S2_Tb_465_lowleft.tif]

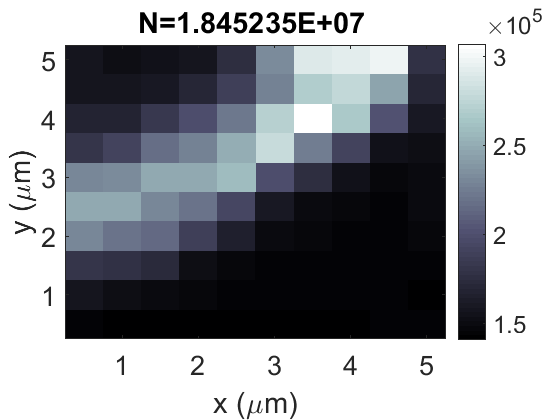

Supplement: S2 File — (ZIP) [file pone.0188359.s002.zip › Images/S2_Tb_488_lowright.tif]

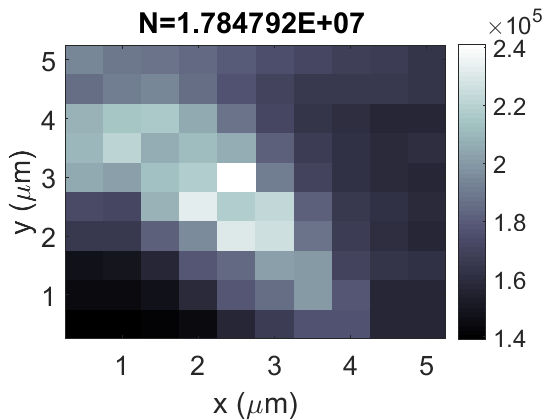

Supplement: S2 File — (ZIP) [file pone.0188359.s002.zip › Images/S2_Tb_560_topright.tif]

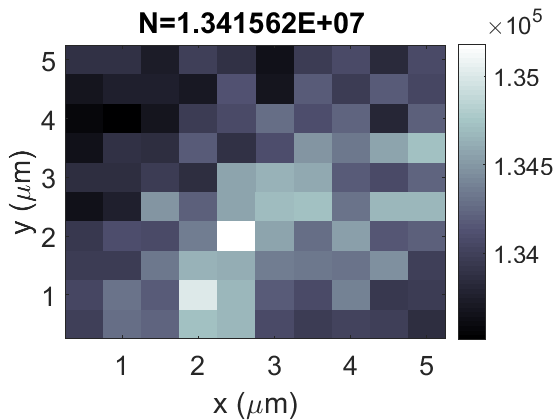

Supplement: S2 File — (ZIP) [file pone.0188359.s002.zip › Images/S2_Tb_633_topleft.tif]

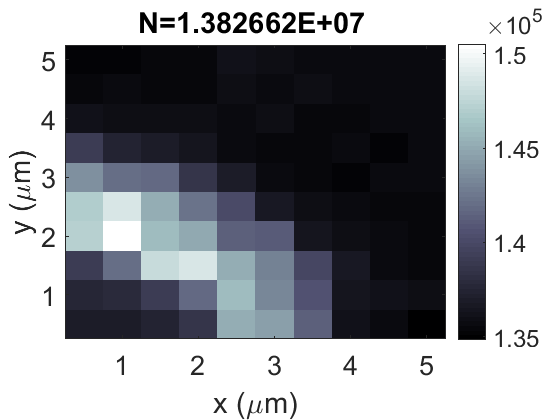

Supplement: S2 File — (ZIP) [file pone.0188359.s002.zip › Images/S3_EuTb_560_topright.tif]

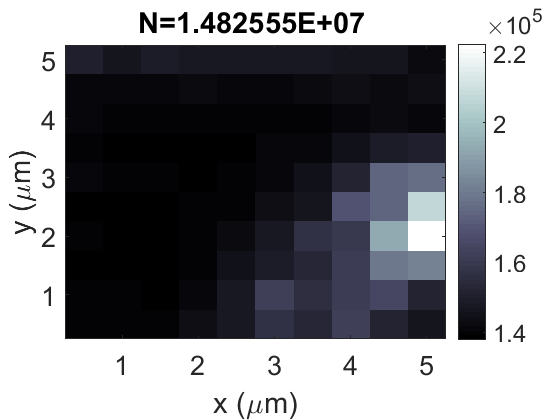

Supplement: S2 File — (ZIP) [file pone.0188359.s002.zip › Images/S3_EuTb_633_topleft.tif]

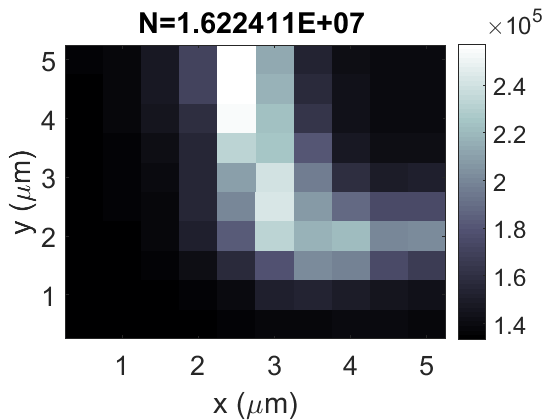

Supplement: S2 File — (ZIP) [file pone.0188359.s002.zip › Images/S3_Eu_465_lowleft.tif]

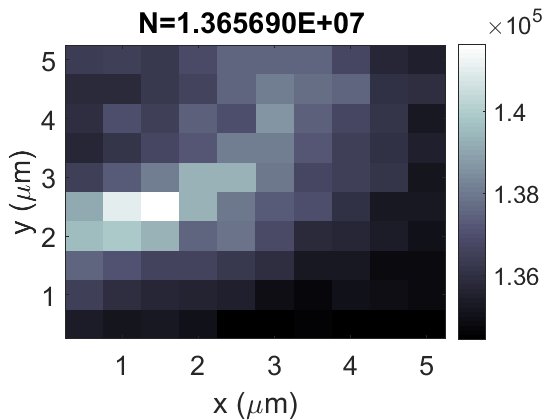

Supplement: S2 File — (ZIP) [file pone.0188359.s002.zip › Images/S3_Eu_488_lowright.tif]

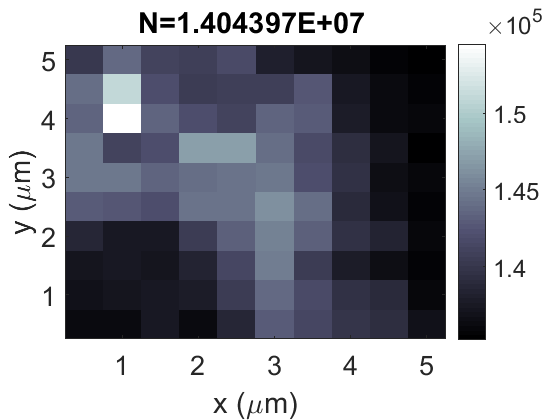

Supplement: S2 File — (ZIP) [file pone.0188359.s002.zip › Images/S3_Eu_560_topright.tif]

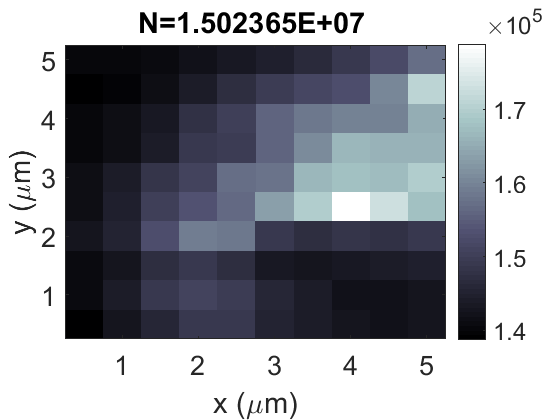

Supplement: S2 File — (ZIP) [file pone.0188359.s002.zip › Images/S3_Eu_633_topleft.tif]

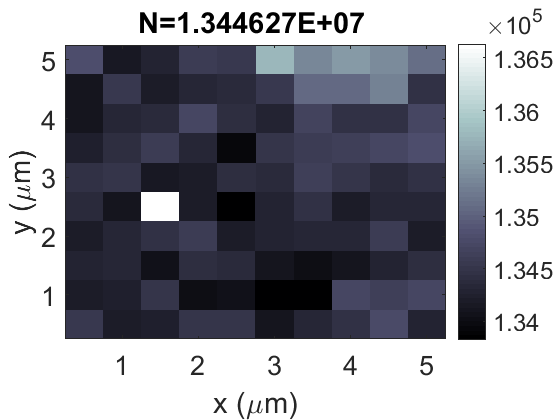

Supplement: S2 File — (ZIP) [file pone.0188359.s002.zip › Images/S3_Tb_465_lowleft.tif]

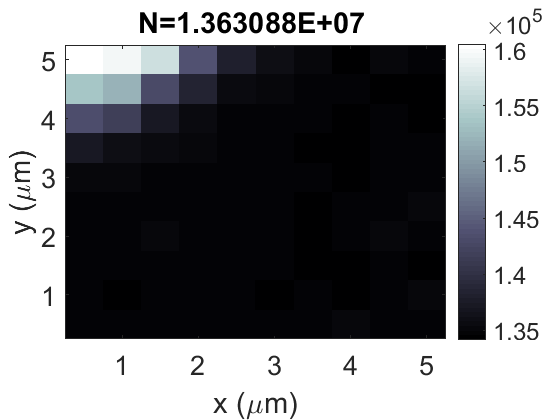

Supplement: S2 File — (ZIP) [file pone.0188359.s002.zip › Images/S3_Tb_488_lowright.tif]

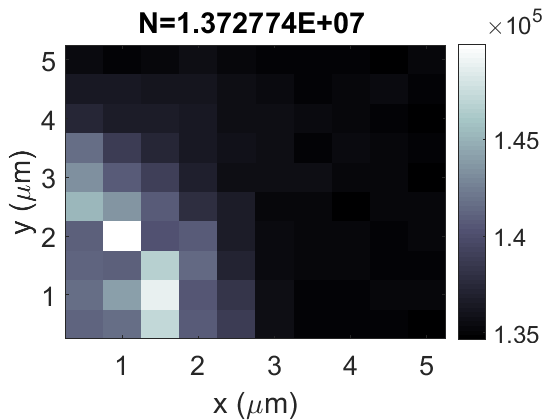

Supplement: S2 File — (ZIP) [file pone.0188359.s002.zip › Images/S3_Tb_560_topright.tif]

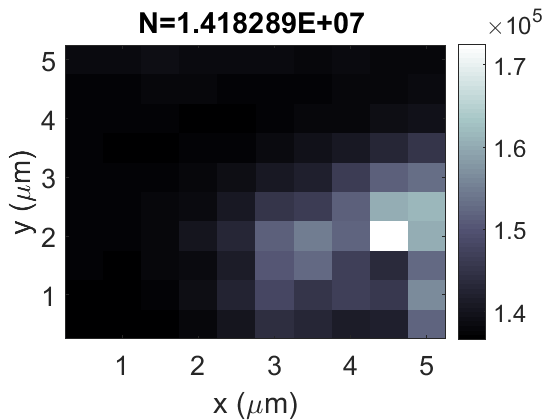

Supplement: S2 File — (ZIP) [file pone.0188359.s002.zip › Images/S3_Tb_633_topleft.tif]

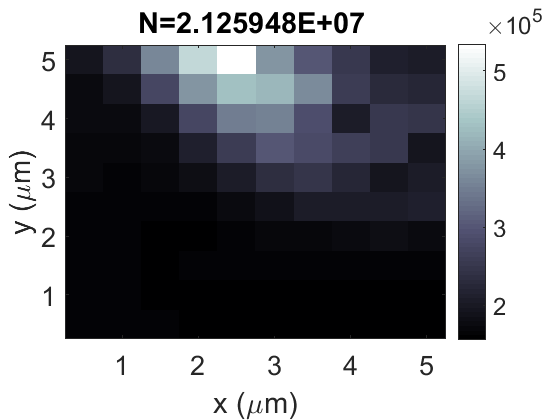

Supplement: S2 File — (ZIP) [file pone.0188359.s002.zip › Images/S4_Eu_465_lowleft.tif]

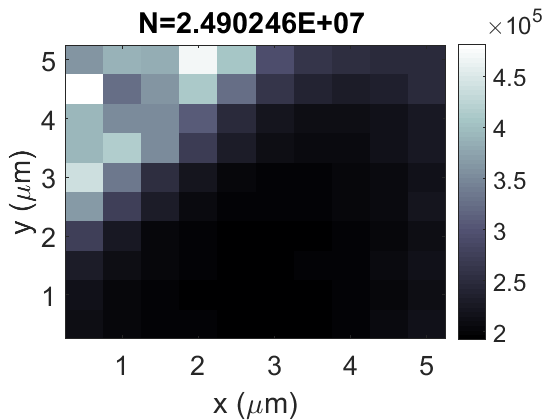

Supplement: S2 File — (ZIP) [file pone.0188359.s002.zip › Images/S4_Eu_488_lowright.tif]

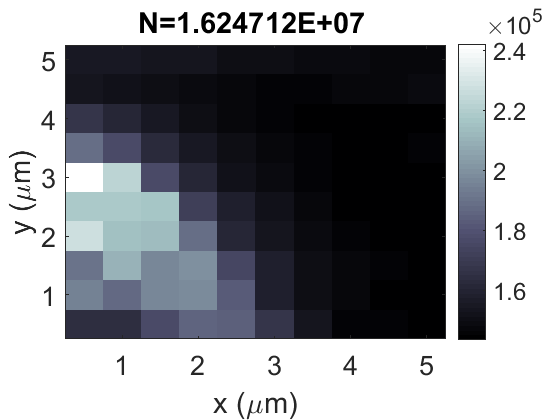

Supplement: S2 File — (ZIP) [file pone.0188359.s002.zip › Images/S4_Eu_560_topright.tif]

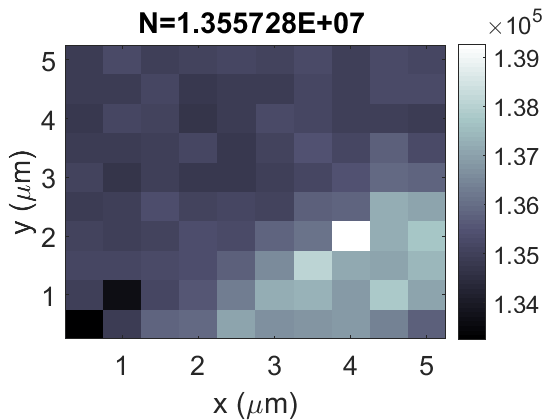

Supplement: S2 File — (ZIP) [file pone.0188359.s002.zip › Images/S4_Eu_633_topleft.tif]

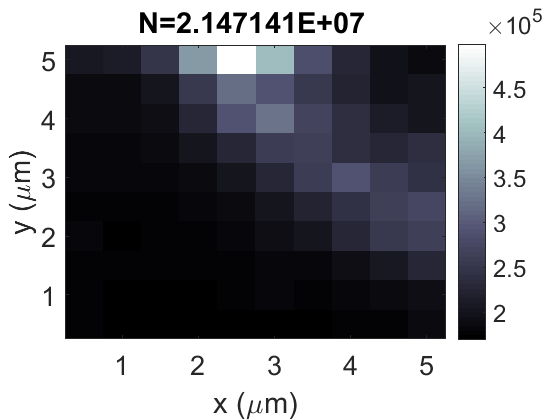

Supplement: S2 File — (ZIP) [file pone.0188359.s002.zip › Images/S4_Tb_465_lowleft.tif]

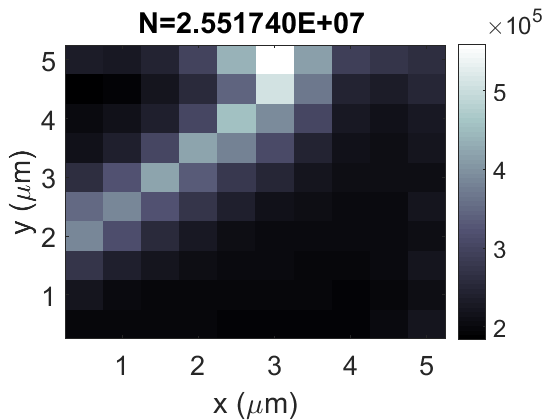

Supplement: S2 File — (ZIP) [file pone.0188359.s002.zip › Images/S4_Tb_488_lowright.tif]

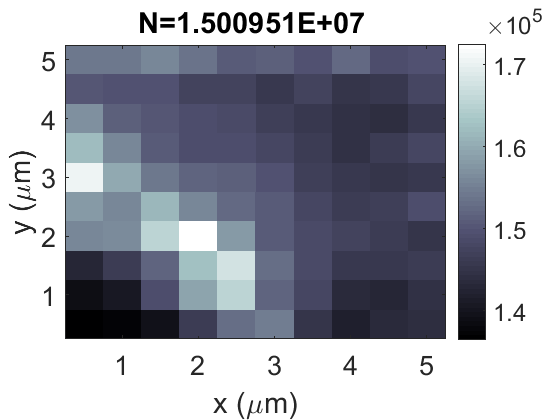

Supplement: S2 File — (ZIP) [file pone.0188359.s002.zip › Images/S4_Tb_560_topright.tif]

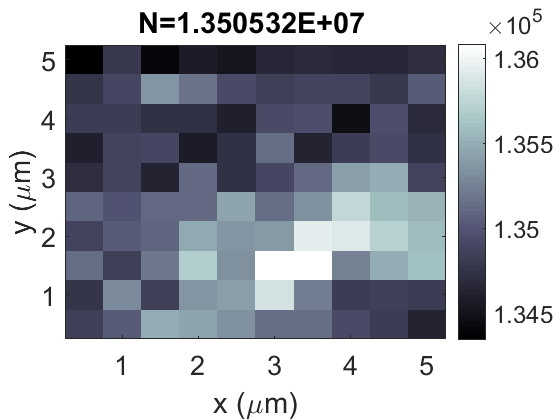

Supplement: S2 File — (ZIP) [file pone.0188359.s002.zip › Images/S4_Tb_633_topleft.tif]

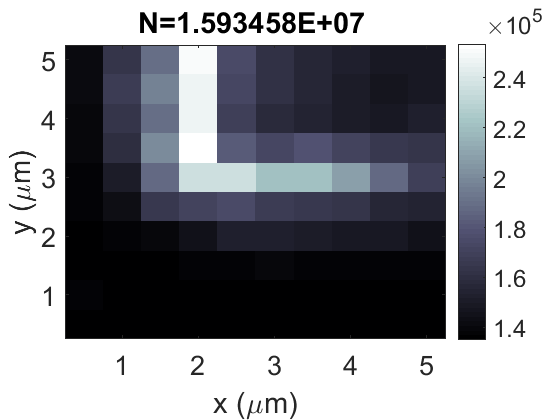

Supplement: S2 File — (ZIP) [file pone.0188359.s002.zip › Images/S5_Eu_465_lowleft.tif]

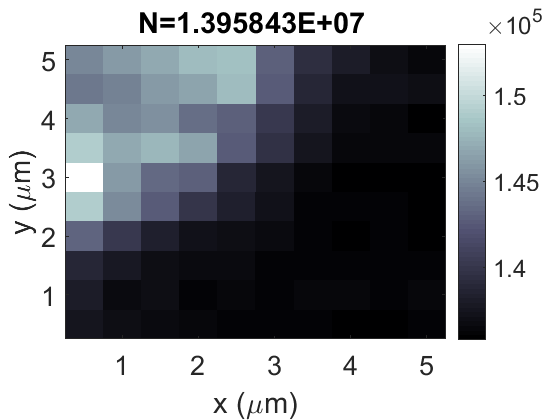

Supplement: S2 File — (ZIP) [file pone.0188359.s002.zip › Images/S5_Eu_488_lowright.tif]

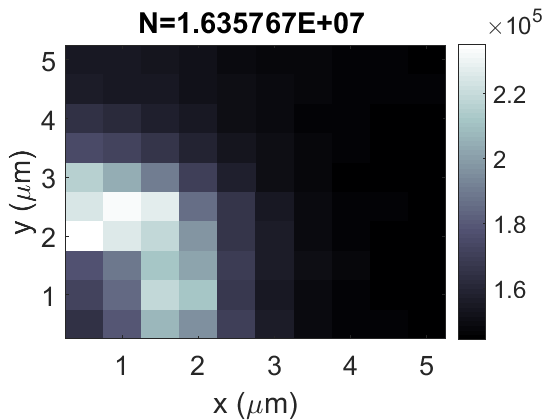

Supplement: S2 File — (ZIP) [file pone.0188359.s002.zip › Images/S5_Eu_560_topright.tif]

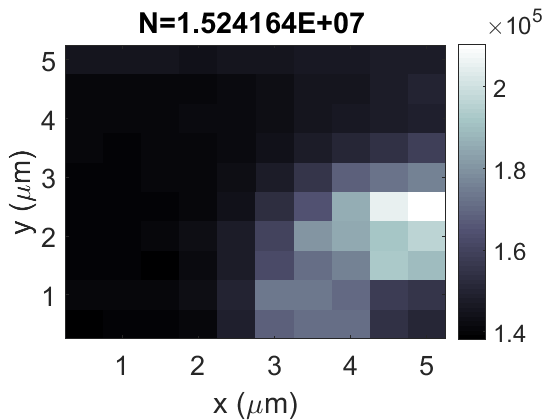

Supplement: S2 File — (ZIP) [file pone.0188359.s002.zip › Images/S5_Eu_633_topleft.tif]

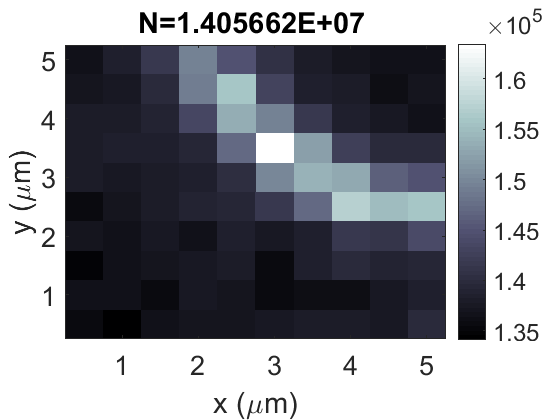

Supplement: S2 File — (ZIP) [file pone.0188359.s002.zip › Images/S5_Tb_465_lowleft.tif]

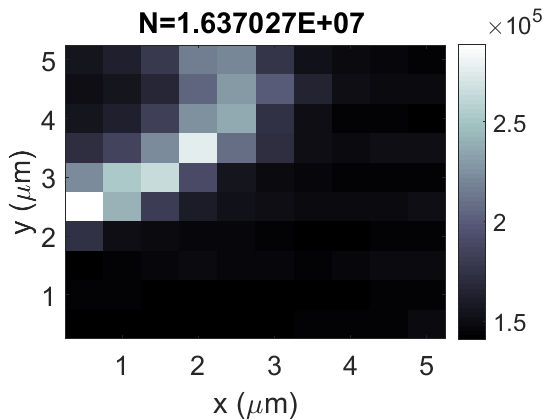

Supplement: S2 File — (ZIP) [file pone.0188359.s002.zip › Images/S5_Tb_488_lowright.tif]

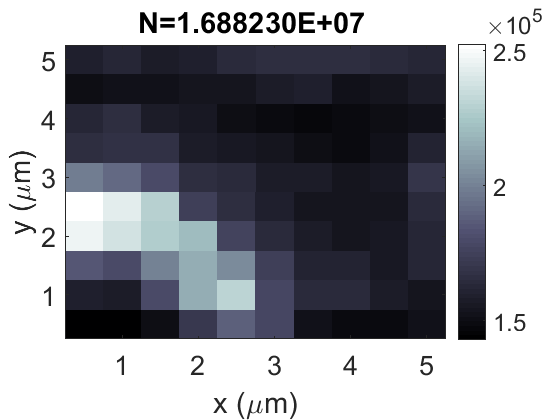

Supplement: S2 File — (ZIP) [file pone.0188359.s002.zip › Images/S5_Tb_560_topright.tif]

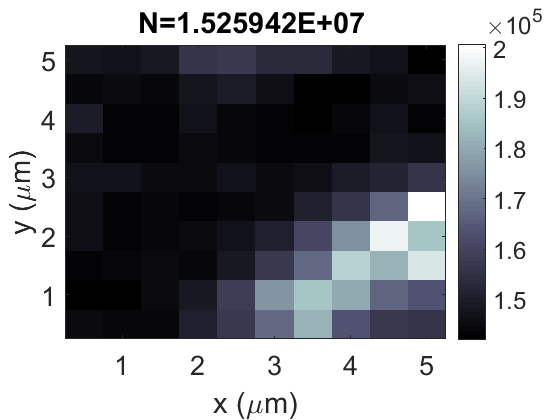

Supplement: S2 File — (ZIP) [file pone.0188359.s002.zip › Images/S5_Tb_633_topleft.tif]

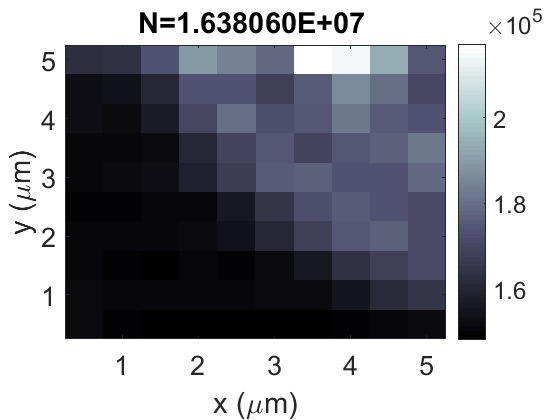

Supplement: S2 File — (ZIP) [file pone.0188359.s002.zip › Images/S6_Eu_465_lowleft.tif]

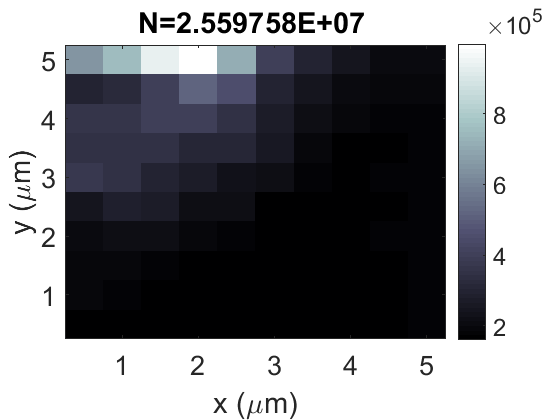

Supplement: S2 File — (ZIP) [file pone.0188359.s002.zip › Images/S6_Eu_488_lowright.tif]

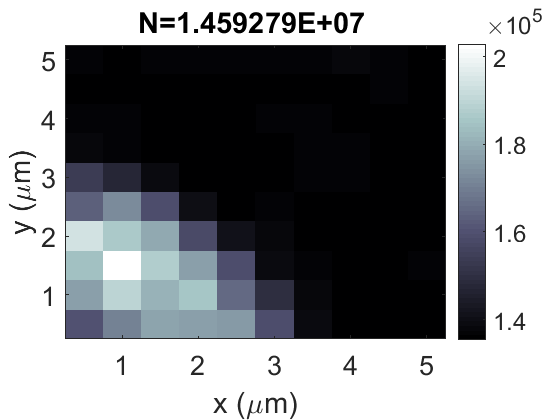

Supplement: S2 File — (ZIP) [file pone.0188359.s002.zip › Images/S6_Eu_560_topright.tif]

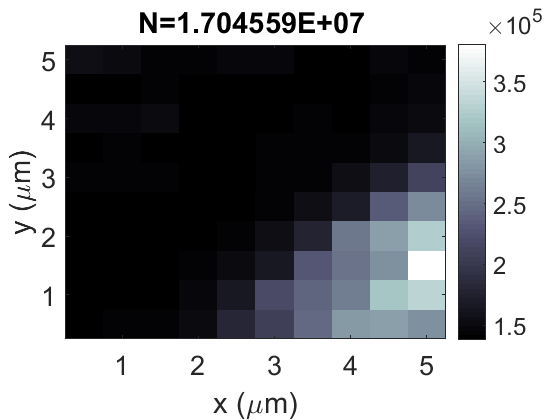

Supplement: S2 File — (ZIP) [file pone.0188359.s002.zip › Images/S6_Eu_633_topleft.tif]

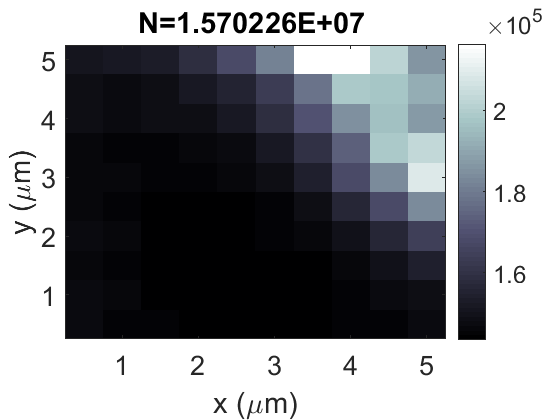

Supplement: S2 File — (ZIP) [file pone.0188359.s002.zip › Images/S6_Tb_465_lowleft.tif]

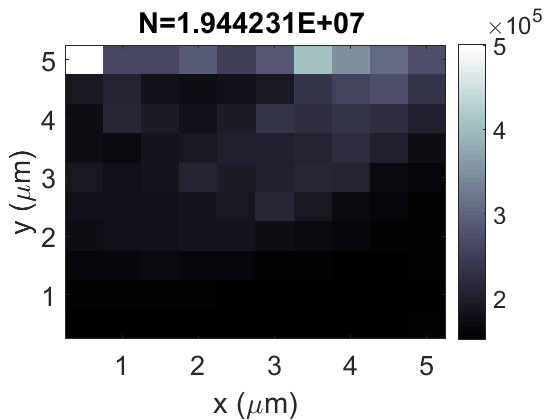

Supplement: S2 File — (ZIP) [file pone.0188359.s002.zip › Images/S6_Tb_488_lowright.tif]

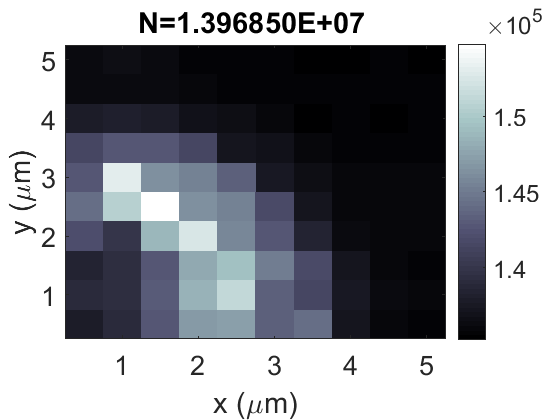

Supplement: S2 File — (ZIP) [file pone.0188359.s002.zip › Images/S6_Tb_560_topright.tif]

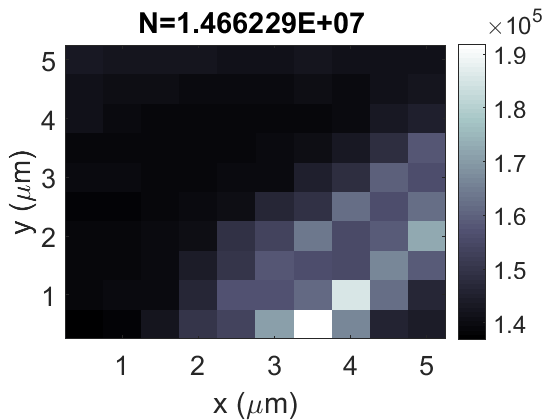

Supplement: S2 File — (ZIP) [file pone.0188359.s002.zip › Images/S6_Tb_633_topleft.tif]

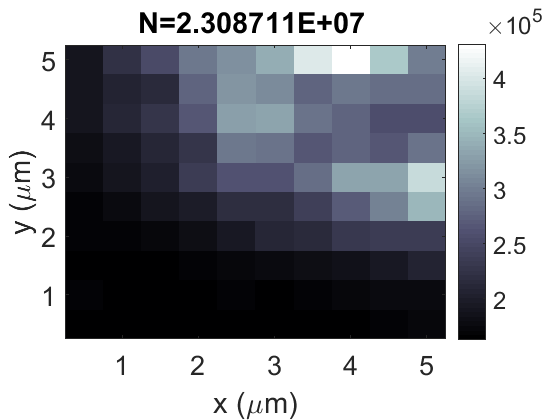

Supplement: S2 File — (ZIP) [file pone.0188359.s002.zip › Images/S7_Eu_465_lowleft.tif]

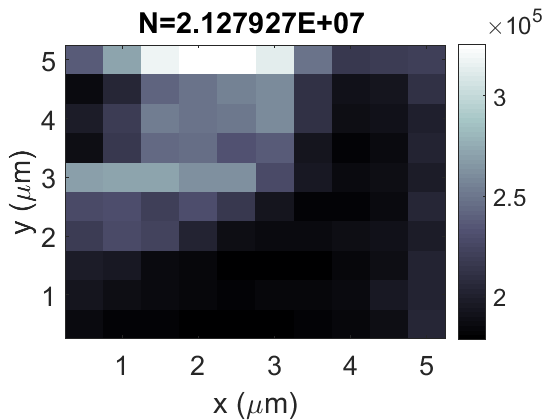

Supplement: S2 File — (ZIP) [file pone.0188359.s002.zip › Images/S7_Eu_488_lowright.tif]

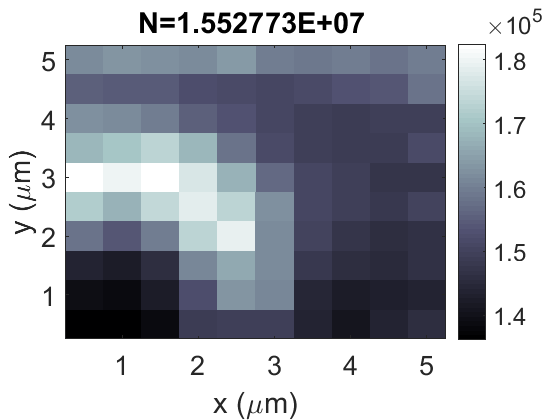

Supplement: S2 File — (ZIP) [file pone.0188359.s002.zip › Images/S7_Eu_560_topright.tif]

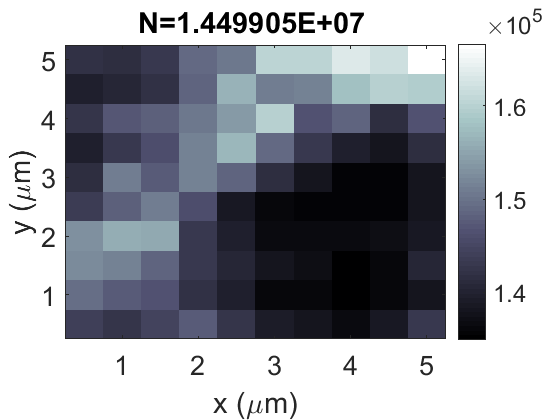

Supplement: S2 File — (ZIP) [file pone.0188359.s002.zip › Images/S7_Eu_633_topleft.tif]

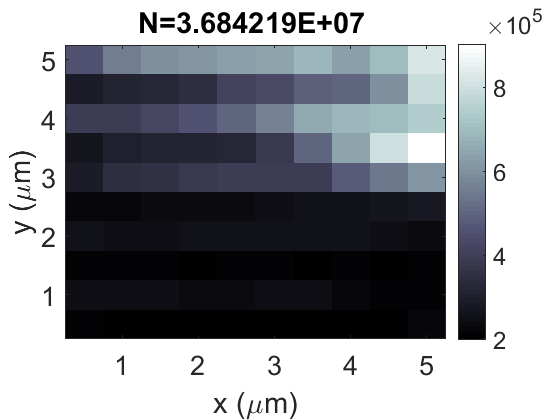

Supplement: S2 File — (ZIP) [file pone.0188359.s002.zip › Images/S7_Tb_465_lowleft.tif]

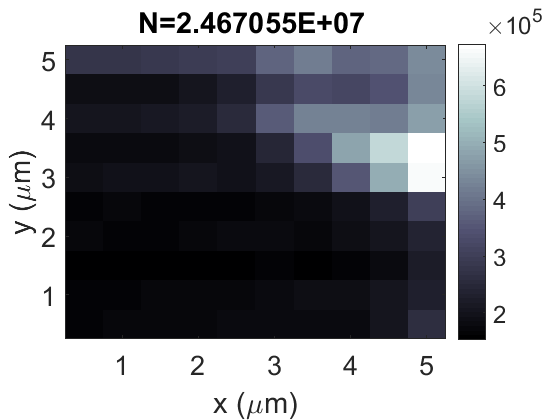

Supplement: S2 File — (ZIP) [file pone.0188359.s002.zip › Images/S7_Tb_488_lowleft.tif]

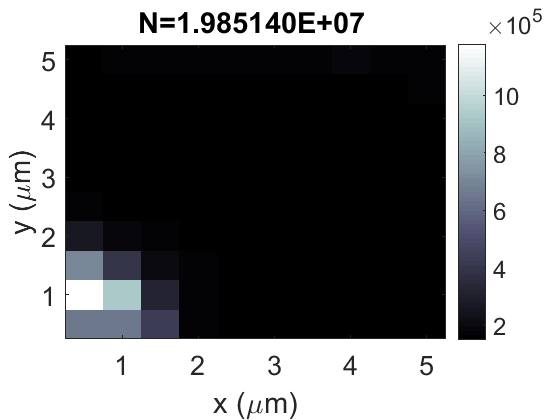

Supplement: S2 File — (ZIP) [file pone.0188359.s002.zip › Images/S7_Tb_560_topright.tif]

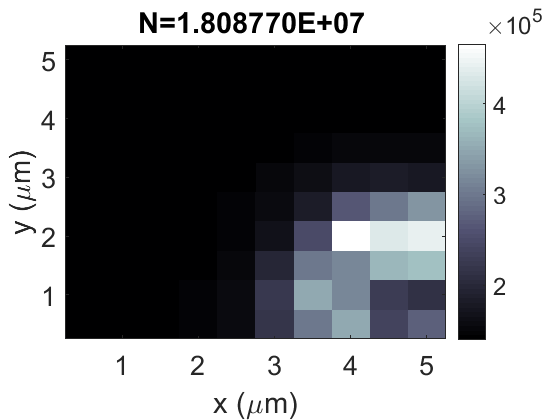

Supplement: S2 File — (ZIP) [file pone.0188359.s002.zip › Images/S7_Tb_633_topleft.tif]
